# Computational simulations of tumor growth and treatment response: Benefits of high-frequency, low-dose drug regimens and concurrent vascular normalization

**DOI:** 10.1371/journal.pcbi.1011131

**Published:** 2023-06-08

**Authors:** Mohammad R. Nikmaneshi, Rakesh K. Jain, Lance L. Munn

**Affiliations:** 1 Edwin L. Steele Laboratories, Department of Radiation Oncology, Harvard Medical School and Massachusetts General Hospital, Harvard Medical School, Boston, Massachusetts, United States of America; 2 Department of Mechanical Engineering, Sharif University of Technology, Tehran, Iran; University of Virginia, UNITED STATES

## Abstract

Implementation of effective cancer treatment strategies requires consideration of how the spatiotemporal heterogeneities within the tumor microenvironment (TME) influence tumor progression and treatment response. Here, we developed a multi-scale three-dimensional mathematical model of the TME to simulate tumor growth and angiogenesis and then employed the model to evaluate an array of single and combination therapy approaches. Treatments included maximum tolerated dose or metronomic (i.e., frequent low doses) scheduling of anti-cancer drugs combined with anti-angiogenic therapy. The results show that metronomic therapy normalizes the tumor vasculature to improve drug delivery, modulates cancer metabolism, decreases interstitial fluid pressure and decreases cancer cell invasion. Further, we find that combining an anti-cancer drug with anti-angiogenic treatment enhances tumor killing and reduces drug accumulation in normal tissues. We also show that combined anti-angiogenic and anti-cancer drugs can decrease cancer invasiveness and normalize the cancer metabolic microenvironment leading to reduced hypoxia and hypoglycemia. Our model simulations suggest that vessel normalization combined with metronomic cytotoxic therapy has beneficial effects by enhancing tumor killing and limiting normal tissue toxicity.

## Author summary

Effective treatment of solid tumors with injected drugs requires that sufficient exposure of cancer cells to the cytotoxic drugs. However, non-uniform and poorly functioning blood vessels make this difficult. The amount of drug that reaches a given cancer cells depends on many factors, including the drug chemistry, its lifetime in the blood circulation, its ability to cross the blood vessel wall and enter the tissue, and the schedule of the injections. We present a mathematical model of tumor growth, angiogenesis, metabolism and drug transport that examines how these processes affect the response to treatment. We find that low dose, high frequency (metronomic) therapy normalizes the tumor vasculature to improve drug delivery and that combining an anti-cancer drug with a drug that specifically enhances vascular function increases tumor killing and reduces drug accumulation in normal tissues. Our model simulations suggest that vessel normalization combined with metronomic cytotoxic therapy has beneficial effects by enhancing tumor killing and limiting normal tissue toxicity.

## Introduction

Cytotoxic drugs kill any cell actively undergoing division, thus often causing significant toxicity in patients. These toxicities limit the dose and scheduling of chemotherapeutics. Thus, patient’s quality of life needs to be weighed against tumor cell killing during treatment. Because of these considerations, alternatives to the commonly- employed maximum-tolerated dose (MTD) schedule have been proposed. One such strategy is to use low-dose, high-frequency “metronomic (M)” regimens that provide sufficient exposure of drug to the tumor while decreasing cytotoxic effects in normal tissues caused by a high plasma concentration just after injection [1–5].

Metronomic therapy has been characterized and tested in multiple preclinical models with the goal of understanding the underlying mechanisms that differ between maximum tolerated dose and metronomic administration [6]. In an orthotopic breast cancer model, metronomic topotecan combined with pazopanib (an antiangiogenic tyrosine kinase inhibitor) prolonged survival, even in the advanced metastatic survival setting by decreasing vascularization and enhancing tumor cell apoptosis. The increased activity with metronomic administration has been attributed to normalization of the vasculature through inhibition of HIF1alpha and ABCG2 [7–9].

Metronomic therapy has been tested in the clinic in gastrointestinal cancers [10]. Metronomic cyclophosphamide with celecoxib in gastrointestinal patients with advanced disease showed low toxicity and increased activity [11]. In PDAC patients, it was shown that metronomic scheduling informed by chemosensitivity analysis improved overall survival [12]. Furthermore, several preclinical and clinical HCC studies suggest that metronomic chemotherapy may provide advantages for treatment of advanced HCC and postsurgical adjuvant treatment of HCC [13].

In patients with metastatic castration-resistant prostate cancer (mCRPC), metronomic therapy has been proposed as a viable alternative strategy, as it is well tolerated in older and frail patients and more affordable than conventional therapies [14]. Metronomic therapy has also been tested in patients with metastatic breast cancer, where it resulted in better outcomes compared to conventional dosing [15]. In head and neck cancers, studies have shown that low-dose methotrexate and celecoxib modulates immune response, angiogenesis and cytotoxic action [16], and maintenance oral metronomic chemotherapy improved overall and progression-free survivals with decreased toxicity in patients with metastatic/recurrent nasopharyngeal carcinoma [17].

In contrast, oral metronomic methotrexate and celecoxib for locally advanced esophageal/GEJ SCC did not improve outcomes and may adversely affect survival rates [18]. Similarly, in small cell lung cancer, low-dose/high-frequency treatment provided no benefit over conventional therapy in terms of response rates, survival, toxicity or other aspects of quality of life [19]. This highlights the need for a fundamental understanding of the mechanisms of beneficial metronomic scheduling, and a more rigorous rationale for applying metronomic regimens. Metronomic chemotherapy dose regimens are generally determined empirically, but the outcomes in terms of efficacy and toxicity can be sensitive to pharmacokinetic parameters [2]. Thus, the proper scheduling of anti-cancer drugs requires a better understanding of drug pharmacokinetics and transport properties in the tumor microenvironment (TME).

Treatment efficacy is influenced by heterogeneities in the tumor microenvironment [20,21]. Structural and functional abnormalities of tumor vasculature hinder drug delivery to cancer cells, especially those far from functional vessels [22–26]. Tumor blood vessels are highly permeable, but abnormally distributed [27–33]. Tumor lymphatic vessels are dysfunctional, and thus unable to drain the interstitial fluid [34–36]. Consequently, interstitial fluid pressure (IFP) is abnormally high in solid tumors [33,37]. Because IFP and intravascular pressures are approximately equal in the tumor, transvascular pressure gradients are not sufficient for convection of drugs from the tumor vessels into the tumor interstitium [33,38–41]. The poor transvascular convection in solid tumors limits the delivery of large molecular weight drugs and nano-formulations that rely on convective transport [27,42].

This lack of fluid homeostasis in tumor tissue causes fluid stagnation, not only in the tissue, but also in poorly-perfused, immature vessels, further limiting transport through the vasculature to deeper regions of the tumor [43]. By blocking major angiogenic signaling pathways such as those involving vascular endothelial growth factor (VEGF), it is possible to induce vascular normalization, thus restoring fluid homeostasis transiently [28]. Previous studies have shown that restoring the balance between pro- and anti-angiogenic signaling can reduce the hyperpermeability of tumor vessels to decrease the interstitial fluid pressure, restoring transvascular and intravascular pressure gradients, improving drug delivery [26,33,41,44–48]. While high doses of antiangiogenic drugs cause vascular pruning and can hinder drug delivery, lower, “normalizing” doses prevent new vessel formation, decrease vessel wall permeability and tumor IFP [28,43,49], and improve drug delivery to the tumor [27,50–52].

Vessel normalization, however, would also increase nutrient delivery to tumor cells. In addition, the survival and proliferation of cancer cells will depend on their exposure to drug, which depends on the distance from a perfused vessel, the drug transport properties and the interval between doses [22]. It is possible that cancer cells reside and survive in regions that are far enough from vessels so that they avoid lethal levels of drug, but still receive sufficient nutrients for a short time [53].

These often-competing considerations of drug delivery, normal tissue toxicity, optimal vascularization and cancer survival confound optimal cancer treatment. Such complex systems can often be addressed with multiscale mathematical modeling. Models that recapitulate physical and biological features of the TME have the potential to deconvolve these disparate treatment effects and provide guidance for optimizing combination therapies. Such computational approaches can complement *in vitro* and *in vivo* studies using animal models.

Computational models of the TME and drug delivery can include important features of the TME that govern the treatment outcome. Generally, computational models of tumor growth and TME dynamics can be categorized into three types including continuous, discrete and hybrid continuous-discrete models. The continuous models of TME are developed to simulate the spatiotemporal distributions of TME agents without considering morphological information of the heterogeneity of cancer [33,54–57]. The discrete models of TME deal with the TME morphological heterogeneity, but usually ignore spatiotemporal distributions of biochemical and biomechanical factors of TME [58–60].

Mathematical models of tumor growth and vascularization vary in scope and application, and include models focused primarily on the extension of the angiogenic vessels and topology of the new vasculature [61], and the influence of fluid shear stress and haptotactic migration [62]. Other models have examined the details of lumenogenesis in the forming network [63]. More complex models have been developed to include multiple length scales, ranging from signaling biochemicals to cells and tissues. These hybrid models generally combine continuum models of biospecies diffusion with agent-based models of cells and vessels to simulate tumor growth and angiogenesis. The advantage of such hybrid models is the natural evolution of spatial heterogeneity, a major determinant of nutrient and drug delivery [41,55,64–70], and can reproduce the shift from avascular to vascular growth [41,69–71]. Such models can also be used to evaluate the effects of treatments that affect oncogenic signaling pathways [72] or the importance of physical interactions with the normal tissue and matrix [73]. In addition, continuum models of tumor growth and angiogenesis have been developed to study delivery of chemotherapeutics applied with different treatment schedules [74].

However, there is still a lack of understanding of how tumor heterogeneities, vascularization, dose scheduling and drug pharmacokinetics interact to determine treatment outcome. To address this question, we developed a multi-scale, three-dimensional mathematical model of the TME for simulating the spatiotemporal dynamics of tumor growth, angiogenesis and transport. We use our model as a computational tool to analyze the outcome of combination therapies, considering anti-cancer killing, normal tissue toxicity, and cancer cell migration and invasion. The model is not tumor site specific, but instead was developed to simulate cancers that rely on angiogenesis, have evolution of necrotic zones and are sensitive to chemotherapies.

The multi-scale model includes molecular, cellular, and tissue-level scales, and recapitulates the TME heterogeneity. We use our model to simulate the effects of a) maximum tolerated dose (MTD) administration of the anti-cancer drug, b) metronomic therapy using an anti-cancer drug, c) co-administration of anti-angiogenic and MTD anti-cancer drugs, d) co-administration of anti-angiogenic and metronomic anti-cancer therapies. We compare the tumor responses to these treatment approaches for multiple clinical schedules identify those that control tumor progression with reduced toxicity.

## Methods

Details of the mathematical model can be found in the S1 Text file. Briefly, our multi-scale model domain represents a 10×10×8 mm region of the tissue and considers cancer cell proliferation and migration on a discrete matrix. We also calculate continuous gradients of oxygen, nutrients (glucose), and carbon dioxide, VEGF, Extracellular matrix (ECM) and Matrix metalloproteinases (MMPs), Angiopoietins- 1 and 2 (ang-1 and -2), and anticancer and anti-angiogenic drugs. Angiogenic blood vessels initiate from an idealized circular “mother vessel” that surrounds the tumor at the mid-plane. Angiogenic sprouts migrate from the mother vessel in a biased random walk toward sources of VEGF and haptotactic factors. Sprout extension depends on endothelial cell proliferation.

At each time step, cancer cells are assigned a vitality, which is determined by local nutrient and metabolite concentrations; cell viability and proliferation depend on this vitality score. Proliferation also depends on the local availability of space, so decreases with high cell density. Cancer cells migrate with a random walk, biased toward oxygen and nutrient gradients, and biased against regions of higher cell density [69,75,76]. Transport of oxygen, nutrients and drugs occurs by convection within the vascular network, and by diffusion and advection through the extravascular tissue. Similarly, drugs, fluid and metabolites can enter blood vessels via convection if the local pressure gradient is appropriate. Low oxygen levels trigger the production of VEGF by cancer cells, and VEGF enhances endothelial proliferation and guides the extension of angiogenic vessels. The blood vessels also respond to fluid forces, increasing their diameter in response to higher shear stress [77,78].

Endothelial permeability is increased by VEGF, so transvascular diffusion of plasma, nutrients and drugs is increased in hypoxic regions. In addition to influencing cancer cell proliferation, increased cell density can mechanically compress angiogenic blood vessels, decreasing perfusion [69]. The anti-angiogenic (anti-VEGF) drug inhibits VEGF-induced endothelial cell proliferation and vessel leakiness. The anti-cancer drug converts proliferating cells (both cancer and endothelial cells) to quiescent, and then necrotic cells.

### Mathematical model

The multi-scale mathematical model of TME includes molecular, cellular and tissue scales.

### 1. Molecular scale

The time-dependent concentration distribution of species in the tumor microenvironment, *c*_*i*_, is governed by Eq 1, which includes convection by interstitial fluid flow, molecular diffusion, and a reaction term, *R*_*i*_. The vascular compartment can be a source or sink for a given soluble species, represented as *S*_*i*_:

$$\frac{\partial c_{i}}{\partial t}+\nabla .(r_{f}u_{ins}c_{i})=D_{i}\nabla^{2}c_{i}+R_{i}+S_{i}$$ (1)

*D*_*i*_ is the diffusion coefficients of species *i*, ***u***_***ins***_ is the interstitial fluid flow (IFF) velocity, and *r*_*f*_ is the retardation factor defined by the ratio of the solute velocity to the interstittial fluid velocity. Species: *i = ac (anti-cancer drug)*, *ag (anti-angiogenic agent)*, *g (glocuse)*, *o*_*2*_ *(oxygen)*, *co*_*2*_ *(carbon dioxide)*, *v (VEGF)*, *a1 (ang-1)*, *a2 (ang-2)*, *m (MMPs)*, *and e(ECM)*. Additional details of the mathematical model of molecular scale can be found in the S1 Text file.

### 2. Cellular scale

#### 2.1. Tumor cells

We implement a modified cellular vitality (*υ*)/cellular energy (*ψ*) model to consider the effects of oxygen, glucose and CO_2_ on TC (tumor cell) phenotypes [69]. Cellular vitality is increased with oxygen and glucose and decreased with CO_2_. Cellular energy represents the available units of ATP and determines the bioactivity of the TCs [69,79]. The mathematical model of coupled cellular vitality and cellular energy is presented in Eqs 2 and 3:

$$\upsilon =\varphi (\frac{c_{o_{2}}}{c_{o_{2}}+c_{o_{2}}^{ch}}+k_{W}).\frac{c_{g}}{c_{g}+c_{g}^{ch}}\exp\left(-5(\frac{c_{co2}}{c_{co2}^{ch}}-1{)}^{4}H_{\left(c_{co_{2}}-c_{co_{2}}^{ch}\right)}\right)$$ (2)

$$\frac{d\psi }{dt}=(k_{a}^{p}\upsilon -k_{a}^{c}\frac{\upsilon }{\upsilon +1}-k_{ac}c_{ac}\frac{\upsilon }{\upsilon +1})H_{\left(\upsilon -\upsilon^{ch}\right)}-k_{q}^{c}\frac{\upsilon }{\upsilon +1}H_{\left(\upsilon^{ch}-\upsilon \right)}$$ (3)

In Eq 2, φ is a proportionality coefficient, $c_{o_{2}}^{ch},c_{g}^{ch}$, and $c_{co2}^{ch}$ are oxygen, glucose, and carbon dioxide characteristic concentrations, respectively [69,80]. $H_{\left(c_{co_{2}}-c_{co_{2}}^{ch}\right)}$ is a Heaviside function to ensure that CO_2_ reduces cellular vitality when it’s concentration, $c_{co_{2}}$, exceeds the characteristic value, $c_{co_{2}}^{ch}$. *k*_*W*_ is a constant to reproduce the Warburg effect, which tends to favor cancer cell metabolism via glycolysis rather than the oxidative phosphorylation, which is the preference of most other cells in the body.

In this model, the TCs with *υ* below *υ*^*ch*^ are assumed to be quiescent and those with *υ* above *υ*^*ch*^ are active [69]. The active TCs need to achieve a characteristic energy, *ψ*^*ch*^, before they can proliferate into two new TCs [69,81,82].

The active TCs produce ATP at a linear rate related to cellular vitality with a proportional coefficient, $k_{a}^{p}$, and also consume cellular energy based on a Michaelis Menten (M-M) model with maximum rate $k_{a}^{c}$ and M-M constant of 1 [65,69]. The quiescent TCs consume ATP according to a M-M model with maximum rate $k_{q}^{c}$ and M-M constant of 1. Quiescent TCs with zero cellular energy are converted to necrotic phenotype. Indeed, quiescent TCs can be converted to an active or necrotic state based on cellular vitality and energy, and active TCs can become quiescent; however, necrotic TCs cannot be converted to the other phenotypes. In this model, the anti-cancer drug is assumed to interfere with DNA repair, thereby reducing cellular energy of the active TCs. According to Eq 3, the effect of anti-cancer drug on TCs is imposed with a M-M model with a drug-dependent maximum rate, *k*_*ac*_*c*_*ac*_, and M-M constant equal to 1.

#### 2.2. Endothelial cells

During angiogenesis, endothelial tip cells (tECs) migrate toward positive gradients of VEGF [69,83–85] and stalk endothelial cells (sECs) migrate into the tECs-generated conduits in the ECM and also proliferate to create lumens of the angiogenic neo-vessels [69,83,86,87]. The death state is also considered for sECs based on VEGF concentration (see Eq 23 in S1 Text). Moreover, the sECs can differentiate into tECs in response to high VEGF concentration and high ratio of ang-2 to ang-1, and thus generate bifurcating branches from the neo-vessel wall [83,88].

### 3. Tissue scale: development of tumor tissue and neo-vessel pathways

In response to high VEGF concentration and VEGF gradients, tECs migrate into the ECM to create pathways for angiogenic neo-vessels. Tumor cells can sense the oxygen- and nutrient-rich regions (regions with high cellular vitality potential) in the tissue as well as cell density in the surrounding tissue. In addition to biochemical agents, we assume that tumor-induced solid pressure presents a resistance to the migration of TCs and tECs [41,69]. New TCs are stimulated to migrate toward the locations with high oxygen and nutrients (which can result in cooption of tumor vessels), low solid pressure (i.e., low viable cell concentration). We assume that newly-divided TCs can displace ("crowd") viable cells, but not necrotic cells. tECs are motivated to migrate toward high VEGF concentration regions and low solid pressure. The tECs cannot penetrate the regions occupied by necrotic TCs. The fibronectin gradient in the ECM caused by TC- and tEC-induced MMPs supports haptotactic migration of TCs and tECs [69]. According to the movement mechanisms of tECs and TCs, the tumor growth and angiogenesis are mathematically modeled as Eqs 4 and 5, respectively.


$$\frac{\partial \rho_{tEC}}{\partial t}=\underset{Randomwalk}{\underset{︸}{D_{tEC}\nabla^{2}\rho_{tEC}}}-\nabla .\left(\underset{chemotaxis}{\underset{︸}{\frac{\beta_{c}}{1+\alpha c_{v}}\rho_{tEC}\nabla c_{v}}}+\underset{Haptotaxis}{\underset{︸}{\beta_{h}\rho_{tEC}\nabla c_{e}}}\right)$$ (4)

$$\frac{\partial \rho_{TC}}{\partial t}=\underset{Randomwalk}{\underset{︸}{D_{TC}\nabla^{2}\rho_{TC}}}-\nabla .\left(\underset{Haptotaxis}{\underset{︸}{\beta_{h}\rho_{TC}\nabla c_{e}}}+\underset{Cooption}{\underset{︸}{\beta_{cop}\rho_{TC}\nabla (\upsilon )}}\right)$$ (5)

*ρ*_*tEC*_ and *ρ*_*TC*_ are respectively tECs and TCs densities, *D*_*tEC*_ and *D*_*TC*_ are respectively diffusivity of tECs and TCs in the interstitium, *α* is saturation coefficient of chemotaxis, *β*_*c*_, *β*_*h*_ and *β*_*COP*_ are weight coefficients of chemotaxis, haptotaxis, and cooption, respectively. Other aspects of the tissue scale including vessel growth and remodeling, vessel deformation, and fluid dynamics of TME have been presented in the S1 Text file. The parameters used for computational results of the mathematical model are listed in Table A in S1 Text.

### Computational implementation

#### Computational domain

**Fig 1:**
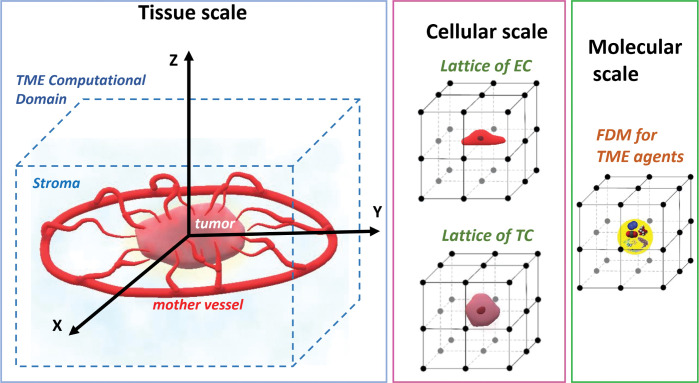
Schematic of TME computational domain with different scales including tissue, cellular, and molecular scales. Vessels sprout form the circular "mother vessel" at the periphery of the domain. A tumor is seeded at the center. The hybrid approach follows the dynamics of cells, vessels, growth factors, oxygen and nutrients in the growing tumor and surrounding normal tissue.

A 10 ×10 ×8 mm cuboid with 201 × 201 × 161 in x, y, and z directions, lattice nodes was selected for the computational domain. The double hybrid continuous-discrete (DHCD) method defined in our previous TME model [69] was applied to solve the mathematical equations of the model. The schematic of TME computational domain with its different scales including tissue, cellular, and molecular scales is shown in Fig 1.

#### Computational method

**Fig 2:**
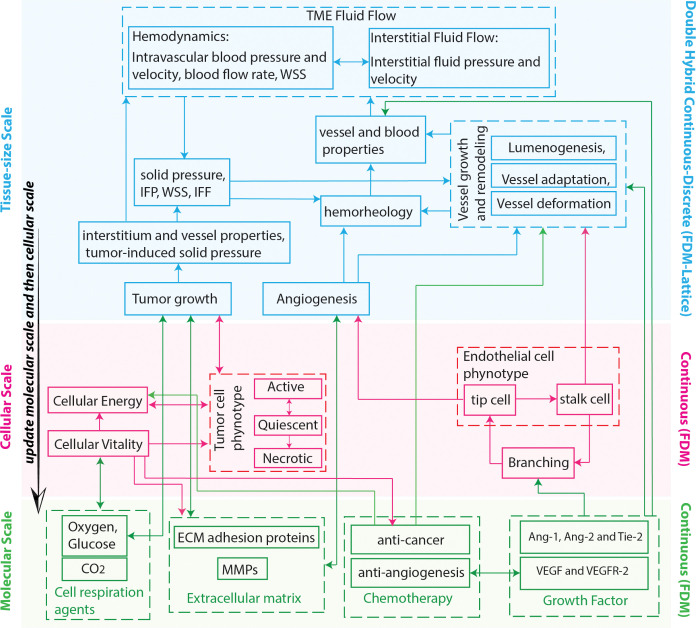
Computational flowchart of the TME model including molecular (green), cellular (red) and tissue-size (blue) scales. The spatiotemporal distributions of molecular agents, along with cellular properties, are calculated within the continuous domain of the model using the finite difference method (FDM). This approach enables the determination of cellular phenotypes and dynamics, which collectively contribute to the formation of tissue-scale accumulations. At the tissue scale, a hybrid continuous-discrete method is employed to integrate the discrete agents, such as tumor and endothelial cells, with the continuous fields of the tissue, including hemodynamics and interstitial fluid flow. This approach enables the simultaneous modeling and interaction of discrete cellular entities within the continuous environment of the tissue.

To solve the governing equations of the TME model, we developed a double hybrid continuous-discrete (DHCD) algorithm. In this computational approach, we divided the model equations into two parts; 1) continuous part and 2) discrete part. In the continuous part of model, the governing equations are numerically solved by an appropriate finite difference method (FDM) on the three-dimensional mesh of the TME cubic domain. The continuous part of model includes the equations of spatiotemporal distributions of the molecular scale, the cellular vitality and energy equations of the cellular scale, the spatiotemporal distributions of biomechanical factors, vessel growth and remodeling. The discrete part of model includes cellular density equations defined for cellular dynamics of tumor and tip endothelial cells [69]. Discrete parts of the model are discretized on two distinct lattices with the same grids as the finite difference mesh applied to the continuous parts. The computational flowchart is shown in Fig 2 to illustrate the relation between different scales of the model and the different computational methods for different scales.

As shown in Fig 2, the computational steps are: 1. Set initial and boundary conditions; 2. Update molecular agents on finite difference mesh (O_2_, glucose, and CO_2_ fields, ECM and MMP fields, VEGF and the VEGF receptor (VEGFR-2) fields, ang-1, ang-2 and their common receptor (Tie-2) fields, anti-cancer and anti-angiogenic drug fields (after treatment)); 3. Update cellular features on the finite difference mesh (cellular vitality and energy fields, probability of branching of vessels (Eq 21 in S1 Text), update phenotypes of tumor and endothelial cells); 4. Update the tissue scale on the finite difference mesh for hemodynamics, interstitial fluid flow, tumor-induced solid stress, and vessel growth and remodeling variables; 5. Update tissue scale on lattice of tumor cells (TCs) for tumor growth; 6. Update the tissue scale on the lattice of endothelia cells (ECs) for angiogenesis; 7. Update the molecular and then cellular scales based on the updated tissue scale information.

## Results and discussion

**Fig 3:**
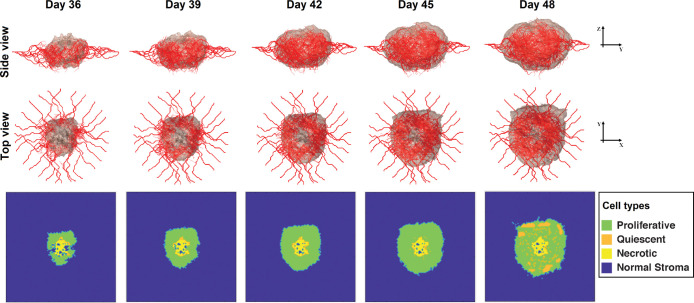
Simulated time course of tumor growth and vascularization without treatment. The side and top views show the three-dimensional tumor and vascular supply for days 36, 39, 42, 45, and 48. The contour plots show the distributions of cell types including proliferative (green), quiescent (orange), and necrotic (yellow) cancer cells along with normal tissue (blue) at the middle cross-section of the domain. We use the well-vascularized tumor from day 36 as the initial condition for testing drug regimens. Stochastic events in the model result in heterogenous tumor growth, angiogenesis and cell phenotype, which influence the delivery and efficacy of drugs.

Because of stochastic processes during angiogenesis and tumor growth, each simulation results in slightly different configurations of the tumor and vasculature. To allow direct comparison between treatments, we simulated tumor growth for 36 days, and then used the resulting tumor as the starting point for all treatment simulations. After 36 days, the tumor is well-vascularized and 4mm in diameter, which is large enough to reveal the effects of different treatments, but small enough so the untreated tumors do not immediately grow beyond the domain boundary. The simulated results of 3D tumor growth and vascularization at different times for an untreated tumor are shown in Fig 3. For each time course, the distribution of cancer cell viability status including proliferative (green), quiescent (orange) and necrotic (yellow) surrounded by normal stroma (blue) at the middle cross-section of the TME domain are also shown.

**Fig 4:**
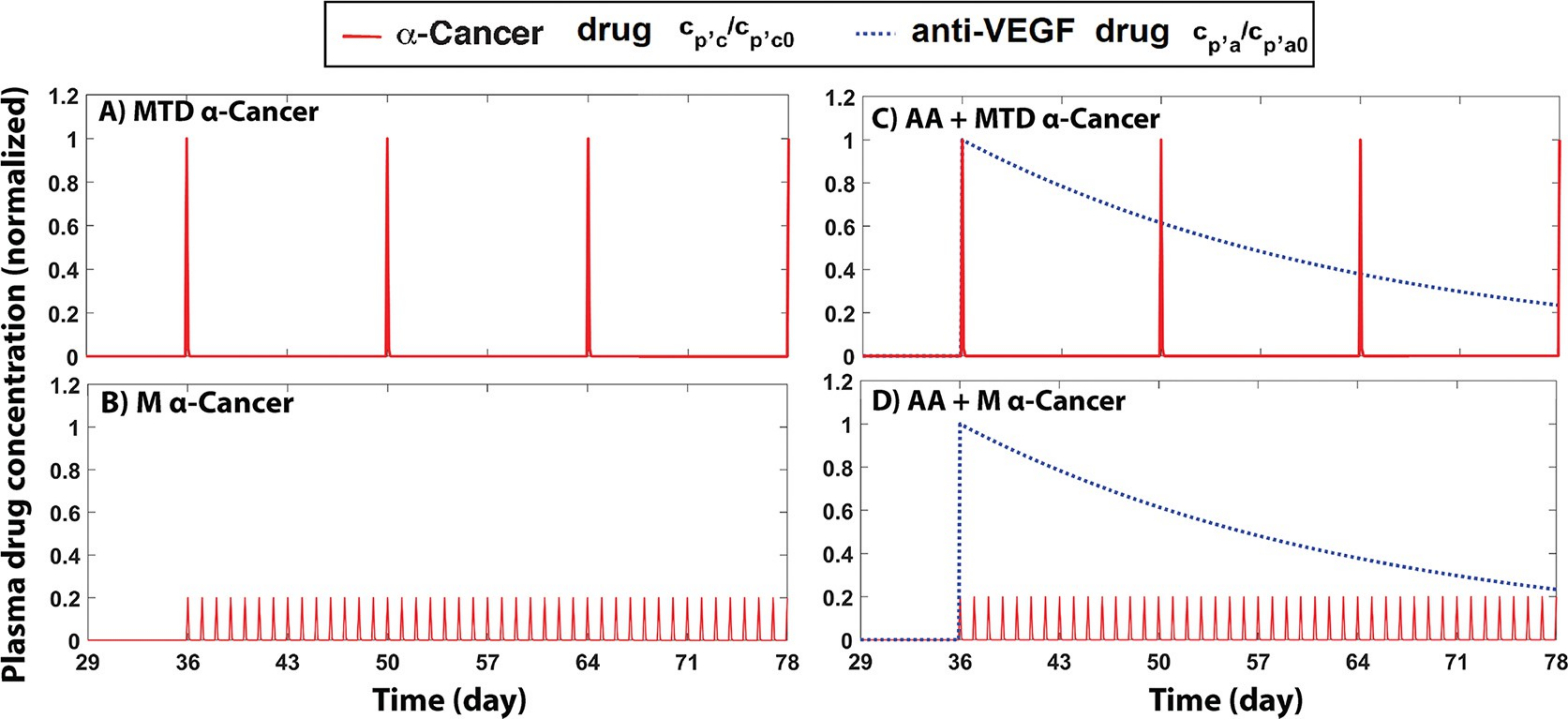
Dose schedules of anti-cancer and anti-angiogenesis drugs. We simulate a maximum tolerated dose (MTD) of the anti-cancer drug, Cisplatin (half-life: 30 min) on day 36 (A), metronomic therapy with Cisplatin, applying daily doses (20% MTD [89]) starting on day 36 (B). We then combined anti-angiogenesis (AA) with MTD (C, “AA+MTD α-Cancer”) or metronomic anti-cancer treatment (D, “AA+M α-Cancer”), both starting on day 36. Red solid lines are the normalized anti-cancer drug concentration in the plasma, and blue dashed-lines are the normalized anti-angiogenic drug plasma concentration.

To compare different treatment schemes, we simulated various single and combination therapy schedules based on clinical protocols of MTD and M treatments with short half-life anti-cancer drugs [89] (Fig 4). A major consideration for treatment design is to limit toxicity to normal tissue, so a hypothetical toxic dose level is indicated by a normalized value equal to 1 in the plots. In these simulations, the anti-cancer drug is Cisplatin with a plasma half-life of 30 min [90] and the anti-angiogenic drug (anti-VEGF antibody) has a half-life of 20 days [91]. Note that for these clinically-relevant regimens, the MTD and metronomic schedules result in different areas under the curve (AUC), with the metronomic schedule being approximately two times greater than the MTD schedule. This likely plays a major role in the observed differences in drug delivery and tumor killing. In Fig A in S1 Text, we further investigate how the AUC of the anti-cancer drug can affect tumor killing and accumulation of drug in tumor and normal tissue.

**Fig 5:**
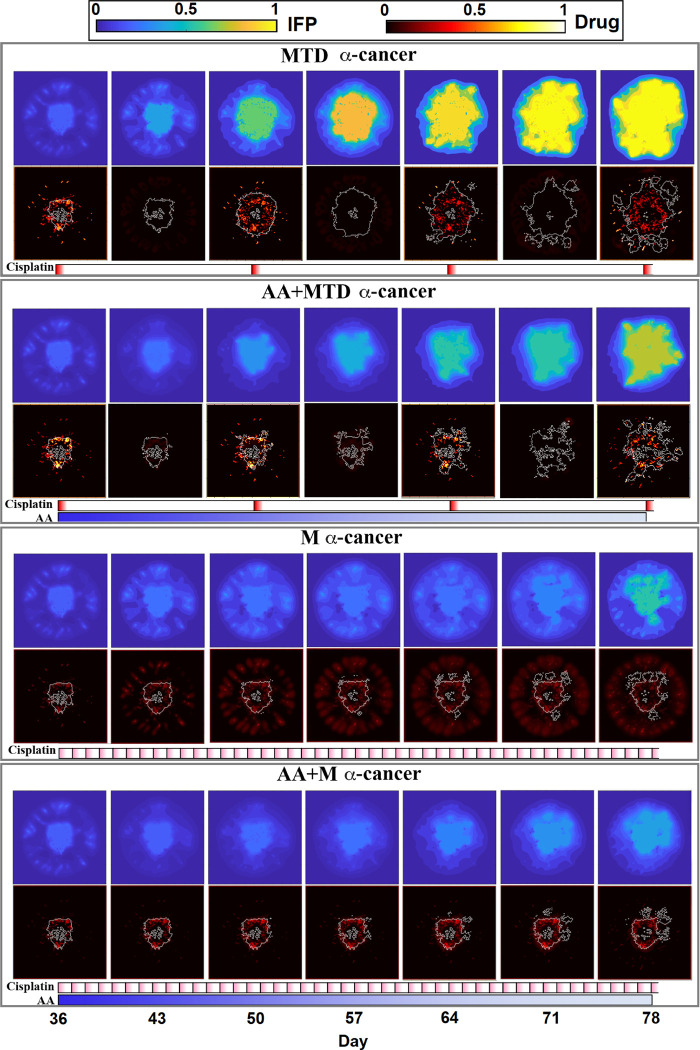
Spatial maps of interstitial fluid pressure (IFP, top panels) and anti-cancer drug distribution in the tumor and surrounding tissue (bottom panels). **The gray lines in the anti-cancer drug maps delineate the tumor.** Treatment with anti-VEGF decreases vessel permeability, preserving the transvascular pressure that drives blood plasma perfusion and interstitial fluid flow. The drug administration schedules are shown below each panel (blue: Anti-angiogenic (AA) drug; red: cisplatin). Note that the metronomic and anti-VEGF treatments can decrease tumor IFP, and that there are differences in drug exposure to the tumor and normal tissues with the various treatments.

The relationships between vessel function, IFP and drug delivery are visualized using spatial maps at different times from day 36 to 78 (Fig 5). Metronomic anti-cancer treatment considerably decreases tumor IFP compared with the MTD treatment, and consequently increases tumor perfusion. Adding anti-VEGF therapy also decreases tumor IFP as expected, increasing tumor perfusion and delivery of the anti-cancer drug into the tumor (comparison between MTD and AA+MTD, and between M and AA+M, Fig 5). MTD alone or when combined with anti-VEGF results in an initially high dose of anti-cancer drug in the tumor, but metronomic application induces a more stable, consistent level of the anti-cancer drug. Combining anti-VEGF, especially with metronomic treatment, decreases drug concentration in normal stroma and increases drug accumulation in the tumor region.

**Fig 6:**
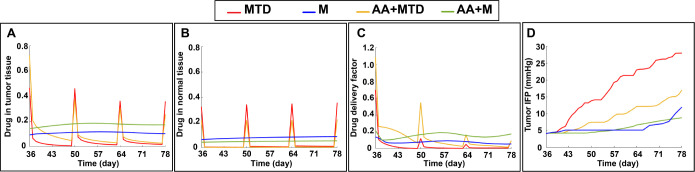
Drug delivery is affected by treatment. Drug concentration in the tumor (A) and normal tissue (B) during different chemotherapy regimens (MTD anti-cancer "MTD," metronomic anti-cancer "M", combination anti-angiogenesis and MTD treatment "AA+MTD", and combination anti-angiogenic and metronomic, "AA+M"). the drug concentrations are normalized with the MTD injection dose. C) Drug delivery factor (calculated by integrating drug concentration over the viable cancer cells over time, normalized by the total number of live cancer cells) for different chemotherapy approaches. D) Spatial average of tumor IFP during different therapeutic regimens.

To quantitatively evaluate the accumulation of anti-cancer drug in the tissue, the spatial average of drug concentrations in tumor and normal stroma for different treatments are calculated in Fig 6A and 6B, respectively. The drug concentrations are normalized with the MTD injection dose. MTD dosing results in large spikes of drug in the tissues, while metronomic therapy induces a more moderate but sustained drug accumulation in the tumor and normal tissue. Adding anti-VEGF therapy decreases drug accumulation in the normal tissue and increases it in the tumor. The shift of distribution due to anti-VEGF is most pronounced when it is combined with metronomic anti-cancer treatment.

To further analyze the efficiency of drug delivery, we defined a drug delivery factor calculated by integrating the anti-cancer drug concentration over only the living cancer cells (i.e. proliferating and quiescent), normalized by the total number of live cancer cells (Fig 6C). Thus, we exclude drug that is localized in necrotic regions. Metronomic therapy, especially when combined with anti-VEGF, results in the most efficient and stable drug delivery to the viable cancer cells (Fig 6C).

Interestingly, the simulations show that metronomic therapy can normalize tumor vessels, decreasing IFP and consequently improving drug delivery to tumor tissue (Fig 6D). Metronomic therapy alone and combined with anti-VEGF results in approximately the same level of IFP reduction. In contrast, adding anti-VEGF to MTD dramatically decreases tumor IFP through vascular normalization. In the model, the vascular normalization by metronomic anticancer therapy is due to two indirect mechanisms. First, the anti-cancer drug kills dividing endothelial cells, effectively inhibiting the formation of new, immature blood vessels. And second, by killing cancer cells, it relieves some metabolic demand and limits hypoxia; this decreases the production of VEGF, consequently reducing angiogenic sprouting and vessel wall permeability.

**Fig 7:**
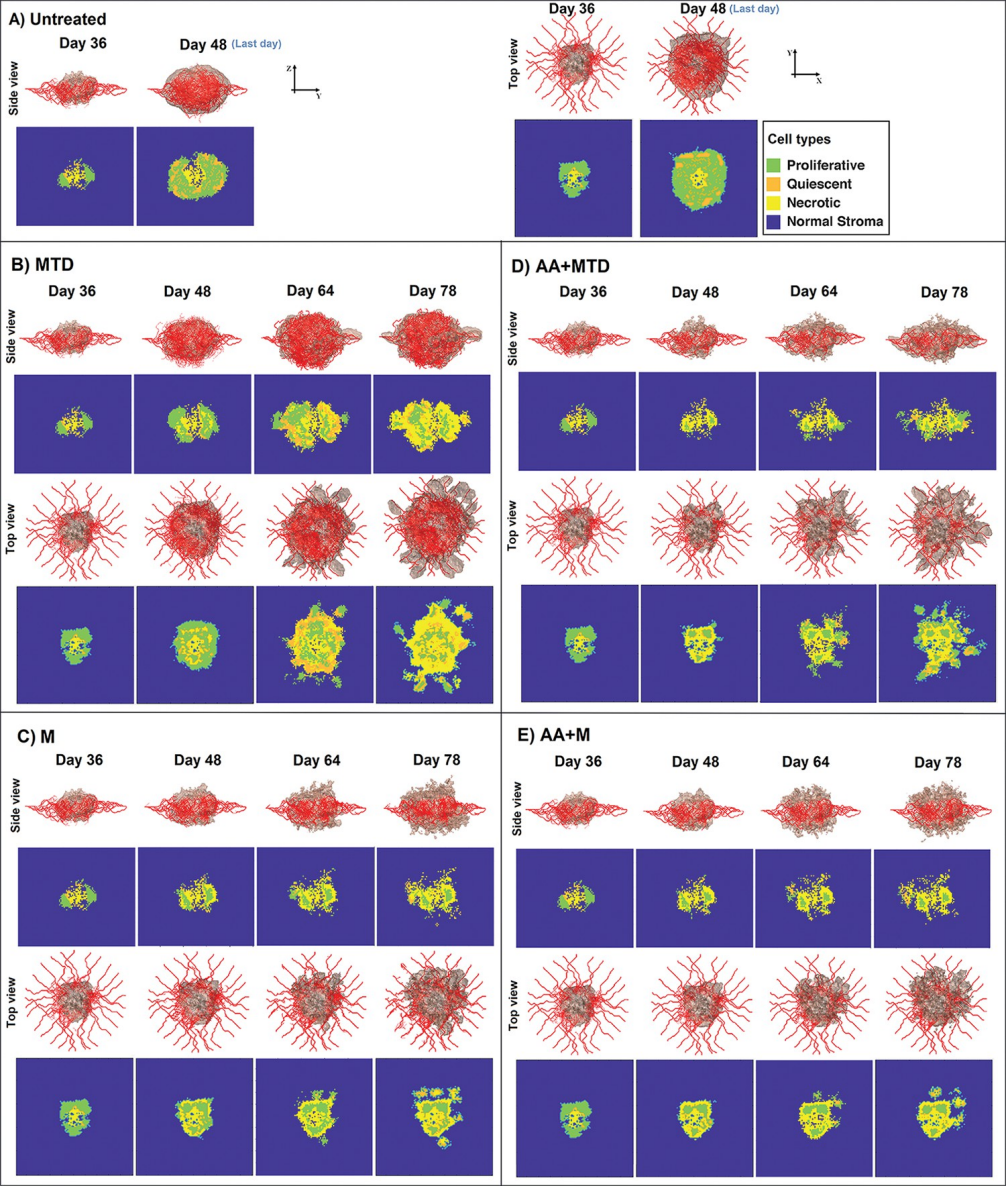
Simulated tumor growth and angiogenesis before and during different treatment regimens. Treatment schedules are detailed in Fig 4) Each top row shows the three-dimensional morphology of the tumor and angiogenic vessels, and the corresponding lower panels show the central cross-section of the tumor in the y-z plane (side view) or x-y plane (top view). Various cell phenotypes are indicated by the contour plot colors: normal tissue around tumor (blue), proliferating cancer cells (green), quiescent cancer cells (orange), and necrotic cancer cells (yellow). The various treatments result in dramatic differences in tumor morphology and viability status.

Because our model is 3D and allows for heterogeneous vascularization and tumor growth patterns, we observe interesting differences in morphology with the various treatment regimens. Fig 7 shows the three-dimensional vascularized tumor and the cellular distributions at different times including the initial day of chemotherapy (day 36), the last day of the untreated (control) tumor (day 48), and the last day for the treated tumors (day 78). The untreated tumor exhibits characteristic invasion patterns resembling the structure of real tumors. It features a semi-central necrotic core (yellow) surrounded by live cancer cells (a mixture of highly proliferative (green) and quiescent cancer cells (orange)) extending away from the central mass, associated with perfused blood vessels. It also shows the stroma regions (blue) encapsulated by cancerous tissue (Fig 7A “untreated”). The untreated tumor adopts the shape of an oblate spheroid due to the higher rate of cell proliferation in regions of high nutrient and oxygen concentrations occupied by blood vessels. In addition, there is preferential migration of the cancer cells toward and around the surrounding vasculature (Fig 7A). This process resembles vascular co-option, a mode of invasion observed in many tumors and a hallmark of glioblastoma malignancy [45,56].

Treatment with the MTD anti-cancer drug slightly decreases tumor size compared with the untreated case at day 48 and creates multi-foci necrosis inside the tumor (Fig 7A and 7B, day 48). MTD treatment has little effect on the vasculature, so nutrients are available even in regions that are becoming necrotic. This allows local regrowth in between doses of the anti-cancer drug, when the drug concentration dissipates. Furthermore, the tumor can expand in the z direction (perpendicular to the plane of the mother vessel), and uninterrupted angiogenesis supplies new vasculature to these regions above and below the tumor to support the growth and invasion. By Day 78, MTD therapy results in a large tumor with a complex morphology that includes a large area of necrosis, many clusters of live cancer cells in the main tumor mass, and multiple groups of viable cancer cells extending into the surrounding normal tissue.

Metronomic treatment produces a more pronounced inhibition of tumor expansion compared with MTD (Fig 7C). Because of the sustained presence of drug in the tumor tissue, there is less opportunity for tumor regrowth (as opposed to MTD), but we still observe multi-foci necrosis and clusters of live cells in the main mass of the tumor and in the surrounding normal tissue. However, unlike MTD, metronomic administration inhibits angiogenesis (sustained drug exposure prevents endothelial proliferation), so the tumor cells invading in the z direction become nutrient-deprived and cannot survive. Therefore, metronomic has advantages over MTD in terms of controlling cancer progression.

When anti-angiogenesis is combined with the anti-cancer drug, angiogenesis is inhibited, vessels are normalized, and nutrients and drugs can penetrate deeper into the tumor. In these cases, for both MTD and metronomic administration, the anti-cancer drug has access to deeper regions of the tumor. However, anti-angiogenesis benefits MTD administration more than metronomic therapy in terms of controlling tumor expansion and invasion (Fig 7B–7D).

**Fig 8:**
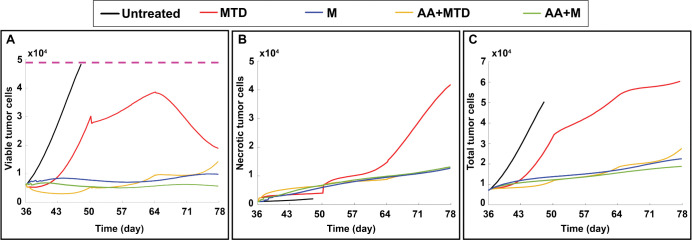
Effect of various treatment regimens on cancer cell viability. A) viable cancer cell number (proliferating plus quiescent), B) number of necrotic cells, C) total number of cancer cells (live and dead) in the tumor. The violet dashed line in panel A shows the maximum allowable tumor size for the simulations; beyond this size, the untreated (control) tumor outgrows the domain at day 48 (the last day of the untreated tumor).

Note that the panels depicting cell proliferation, quiescence and necrosis in Fig 7 are single cross sections in the x-y or y-z planes. Because these tumors are very heterogeneous, a more comprehensive analysis of viability is needed to assess how the drug regimens are performing. To do this, we plotted the viable cancer cells, the necrotic cells and the total number of cancer cells for each case (Fig 8). With each dose of MTD, cancer cells are killed, but this is limited by poor drug penetration. Also, the tumor re-grows in the periods between doses, when intratumor drug concentration is low (red line). Eventually, many of these proliferating cells are also killed, leading to the largest extent of necrosis in the MTD case. Counterintuitively, the simulations predict a large reduction in viable cancer cells after the third dose of MDT therapy (starting on day 64). This is because the model assumes that the existing vessels do not regress as the viable tumor shrinks, and there is an excess of vasculature relative to live cancer cells in this later stage. This increases the amount of drug available for the remaining cancer cells. This prediction requires further exploration, as it is likely that the decrease in metabolic demand caused by cancer cell depletion would be accompanied by pruning of vasculature, and the decrease in tumor size after day 64 would be less pronounced.

Adding anti-angiogenic therapy to MTD has a large effect on cancer cell killing. But again, the tumor is able to regrow in between doses, and cancer cell proliferation out-paces the effect of the treatment (Fig 8A, yellow). Metronomic application produces a more stable suppression of viable cancer cells, and the addition of the anti-angiogenic drug further improves the response (Fig 8, green and blue). Compared with the combination of AA+M, there are ~2-fold more viable cancer cells at day 78 for M treatment alone, ~3-fold more for AA+MTD, and ~4-fold more for MTD alone (Fig 8A).

**Fig 9:**
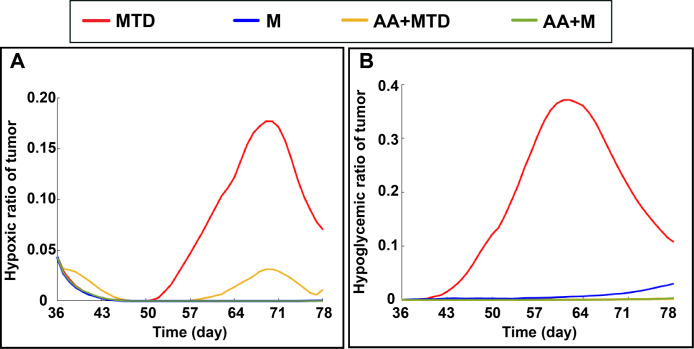
**Hypoxic ratio (A) and hypoglycemic ratio (B) of the tumor under different chemotherapy approaches (MTD, M, AA+MTD, AA+M).** MTD therapy results in the largest variation in hypoxia and hypoglycemia in the tumor because of transport limitations in the TME.

As the model also includes mechanisms involved in tumor cell metabolism, we can analyze differences in hypoxia and glucose levels of tumor induced by the various treatments. In Fig 9 the results of hypoxic and hypoglycemic ratios of tumor for different treatments are shown. MTD treatment results in high levels with the largest variation of hypoxia and hypoglycemia (red) because of transport limitations in the heterogeneous TME. Because of the effects of vascular normalization, adding AA to MTD alleviates these effects considerably (yellow). Metronomic treatment with and without anti-angiogenic therapy eliminates hypoxia and dramatically decreases hypoglycemia (blue, green).

The various treatment approaches also affect cancer cell invasion. To investigate this, we analyzed the migration of cancer cells in the z direction and x-y plane by plotting the distance of the farthest viable cancer cell from the tumor center in these directions (Fig B in S1 Text). The results show that cancer cell invasion in the z direction (Fig B- i in S1 Text) is approximately the same for all treatments, and is less extensive than invasion in the x-y plane (Fig B- ii in S1 Text). Furthermore, because of the increased regrowth and invasion in the intervals between doses, invasion in the x-y plane is much higher for the MTD regimens. This process, which may be related to vessel cooption, results in tumors that have lower aspect ratios (Fig B-iii in S1 Text), as the tumor grows preferentially toward the vasculature. The addition of anti-angiogenic therapy to either MTD or metronomic administration of the anti-cancer drug has little effect on cell invasion.

**Fig 10:**
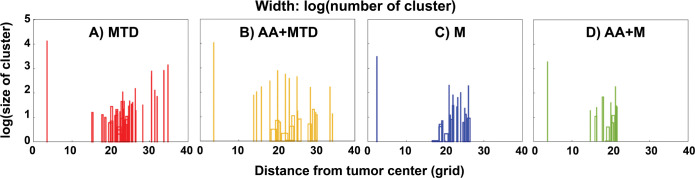
**Distribution of tumor clusters with different sizes at day 78 (number of cells in each cluster) for tumors treated with MTD (A), AA+MTD (B), M (C), AA+M (D).** The width of each bar is proportional to the number of clusters of that size at a given location. Note the larger dispersion of the clusters in A and B, and the more localized clustering in C and D.

Beyond just overall asymmetry and cancer cell invasion, we can also analyze how various cell clusters grow and invade. To do this, we classified each connected cluster of viable cancer cells according to its size (the number of contained cells) and location. We then plotted the size of each cluster vs. the distance from the center of the tumor (Fig 10A–10D).

The distributions of tumor clusters show that MTD treatment alone and MTD combined with anti-angiogenesis result in a large range of tumor cluster sizes distributed over a range of distances away from the tumor center (Fig 10A and 10B). Metronomic treatment decreases the size of the clusters relative to MTD and AA+MTD, and concentrates the tumor clusters to a region closer to the tumor center (Fig 10C). Adding anti-angiogenesis to metronomic treatment further decreases cluster heterogeneity and distance from the tumor center (Fig 10D).

## Discussion

Tumor heterogeneity, inefficient drug delivery, abnormal vasculature and drug pharmacokinetics all have important effects on cancer cell killing and tumor invasion and can be most easily analyzed using a comprehensive, mechanistic model. To this end, we developed a multi-scale, three-dimensional mathematical model of the TME to elucidate how various treatment strategies using anti-cancer and anti-VEGF drugs affect treatment efficacy. By explicitly including blood vessels and stochastic growth, we analyze the spatiotemporal evolution of transport limitations and drug distribution.

Previous studies have reported that metronomic therapy induces vascular normalization. Reports also show that thrombospondin-1 (TSP-1) is upregulated by metronomic therapy, and suggest that this is the mechanism for the observed changes in vascular function. In this work, we observed vessel normalization with metronomic therapy in the absence of TSP-1 production. Indeed, our simulations show that metronomic chemotherapy can affect vascular normalization through chronic suppression of endothelial proliferation, which prevents the creation of new, leaky vasculature.

Our model is an approximation of tumor growth and response to treatment and has several limitations. Because we were interested in exploring transport barriers to tumor treatment, we did not include many biological features of tumors that may also play a role in tumor growth and regression. These include fibroblasts and immune cells that contribute to fibrosis or anti-tumor immune responses. We also neglect potential changes in drug pharmacodynamics due to cancer cell quiescence or tumor hypoxia/hypoglycemia. We would expect, however, that drug efficacy should be better when the tissue is less hypoxic, so the differences seen here would be enhanced with the inclusion of such a mechanism. Since all the model parameters of cancers are not available, the model is not tumor site specific, but instead was developed to simulate cancers that rely on angiogenesis, have evolution of necrotic zones and are sensitive to chemotherapies. However, the tumor microenvironment and its treatment response can be influenced by the tumor location [92]. In addition, our “cellular energy” in Eq 20 varies with time at a rate that depends linearly on the anti-cancer drug concentration. When cellular energy reaches zero, cell death occurs. However, in-vitro studies show that exposure to a low drug concentration for an extended period is less effective in cell killing than a higher concentration for a shorter period, even for the same area under the curve dosage. This would suggest that killing rate depends on dose and AUC in in a more complex way than assumed here. A comparison of model predictions with experimental measurements and other computational simulations is presented in Fig C in S1 Text. To predict the change of output values in response to varying model parameters, a parameter sensitivity analysis was performed (Fig D in S1 Text).

In this study, we focused on a single anti-angiogenic agent (anti-VEGF antibody) and a single anti-cancer drug (Cisplatin). Because the drug circulation and clearance times are critical determinants of delivery and accumulation, drug pharmacokinetics must be considered carefully when developing metronomic or combination therapies. In general, anti-cancer drugs with shorter half-lives yield greater therapeutic benefit when delivered metronomically, because extended periods between doses allows the tumor to recover. Indeed, comparing Cisplatin (plasma half-life = 30 min) with a putative drug with longer half-life (3 hr) shows less difference between MTD and metronomic scheduling (Fig E in S1 Text). However, even for drugs with slower clearance, metronomic therapy may have advantages in terms of lower toxicity, as the systemic plasma distribution does not experience the large spikes associated with MTD administration.

Our simulations demonstrate how blood-borne nutrients and drugs influence the spatiotemporal morphology of the tumor. The tumor expands around the nutrient-rich vasculature surrounding the untreated tumor, as cancer cells migrate toward nutrient gradients and proliferate faster in higher nutrient environments. When treated with anti-cancer drugs alone, the poorly functioning vasculature restricts delivery mainly to the tumor periphery, while the addition of optimal doses of anti-angiogenic therapy leads to better drug penetration into the tumor and consequently more killing of peri-vascular cancer cells.

The simulations also show that the treatment modality can influence the overall shape of the tumor and the number of distinct viable clusters of intratumor and invading cancer cells. The number and size of the remaining clusters of viable cells are related to ability of the drug to penetrate the tissue and distribute throughout the tumor [22]. Thus, cells located far from perfused vessels can survive and proliferate. Our simulations demonstrate that vessel normalization can extend drug penetration, reaching more cancer cells and reducing the number of surviving clusters.

Taken together, our results show that normalizing doses of anti-angiogenic therapy can benefit both MTD and metronomic anti-cancer drug regimens. Furthermore, for drugs with rapid clearance rates, metronomic administration creates sustained drug concentrations in the tumor and prevents extensive regrowth between doses. We find that the addition of anti-angiogenic treatment decreases accumulation of drug in the normal tissue, while enhancing delivery to the tumor and reducing the number of viable tumor clusters. Our model provides a computational platform for exploring these issues of drug delivery and tumor heterogeneity and suggests that metronomic regimens and combination treatments with vascular normalization agents can provide significant benefits for tumor treatment.

## Supporting information

S1 Text Supporting Information for Computational simulations of tumor growth and treatment response: benefits of high-frequency, low-dose drug regimens and concurrent vascular normalization. (PDF)
