# Supplementary material for: Computational simulations of tumor growth and treatment response: Benefits of high-frequency, low-dose drug regimens and concurrent vascular normalization
Source: PLoS Comput Biol. 2023 Jun 8;19(6):e1011131. doi: 10.1371/journal.pcbi.1011131 (PMC10249820; doi:10.1371/journal.pcbi.1011131)
Supplement: S1 Text — (PDF) [file pcbi.1011131.s001.pdf]

Supporting Information for

Computational simulations of tumor growth and treatment response: benefits of high-frequency, low-dose drug regimens and concurrent vascular normalization

Mohammad R. Nikmaneshi[1,2], Rakesh K. Jain[1], Lance L. Munn[*1]

[1]Edwin L. Steele Laboratories, Department of Radiation Oncology, Massachusetts General Hospital, Harvard Medical School, Boston, Massachusetts, 02114, United States of America.

[2]Department of Mechanical Engineering, Sharif University of Technology, Tehran, Iran.

[*]Correspondence and requests for materials should be addressed to **L.L.M.** (email: munn@steele.mgh.harvard.edu).

This file includes:

- **Description of the mathematical model of the tumor microenvironment**
- **The effect of area under the cure (AUC) of anti-cancer drug on tumor expansion**
- **The effect of different treatments on tumor expansion morphology**
- **Validation of model predictions with experimental observations**
- **Parameter sensitivity analysis**
- **Anti-cancer drug pharmacokinetics affect metronomic therapy**
- **Table A – Model Parameters**
- **References**

Description of the mathematical model of the tumor microenvironment

Computational recapitulation of tumor therapy requires a comprehensive mathematical model able to simulate important dynamics of the TME, including spatiotemporal distributions of biochemical and biomechanical factors, relationships between different spatial scales including molecules, cells and tissue. The model should also reproduce morphological heterogeneity of tumor growth and vasculature to properly analyze drug and nutrient distributions. Continuous, discrete and hybrid continuous-discrete models have been previously developed to simulate the TME. Continuous models can predict spatiotemporal distributions of drugs and biomolecules within the TME but ignore the morphological heterogeneity [1-5]. Discrete models can be used to analyze tumor vascularization and growth but don't explicitly consider transport of drugs or biomolecules, or their dynamic distributions [6-8]. Hybrid models combine the advantages of discrete and continuous models and are able to accurately recapitulate many aspects of TME dynamics and heterogeneities [3, 9-13]. Our three-dimensional multi-scale mathematical model of the TME combines discrete and continuous methods to simulate the dynamics of tumor growth, angiogenesis and transport.

1 Molecular scale

The time-dependent concentration distribution of species in the tumor microenvironment, c_i, is governed by Eq. 1, which includes convection by interstitial fluid flow, molecular diffusion, and a reaction term, R_i. The vascular compartment can be a source or sink for a given soluble species, represented as S_i:

$$\frac{\partial c_i}{\partial t} + \nabla.(r_f\,\boldsymbol{u}_{ins} c_i) = D_i \nabla^2 c_i + R_i + S_i \tag{1}$$

D_i is the diffusion coefficients of species *i*, $\boldsymbol{u}_{ins}$ is the interstitial fluid flow (IFF) velocity, and r_f is the retardation factor defined by the ratio of the solute velocity to the interstittial fluid velocity. Species: *i = ac (anti-cancer drug), ag (anti-angiogenic agent), g (glocuse),* o_2 *(oxygen),* co_2 *(carbon dioxide), v (VEGF), a1 (ang-1), a2 (ang-2), m (MMPs), and e(ECM).*

Vessels as a source or sink for soluble species

For transvascular exchange of plasma and soluble species, we used a modified pore model to consider the convectional and diffusional transmigration of particle across the angiogenic vessels [14-18]:

$$S_i = (1-\sigma_{d,i}) S_V \left(\frac{d_v}{d_c}\right)\left[L_p (p_{lum} - p_{ins} - \sigma_v (\pi_{lum} - \pi_{ins}))\right] c_{p,i} \qquad (2)$$
$$+\kappa_i \; S_V \left(\frac{d_v}{d_c}\right)(c_{p,i} - c_i)\frac{P_i}{e^{P_i}-1}$$

In this model, $\sigma_{d,i}$ defined in Eq. 3, is a function of particle size of solute, d_i, and pore size of porous media, d_p, is colloid osmotic (oncotic) reflection coefficient for solute into plasma and σ_v is average oncotic reflection coefficient of plasma proteins, d_v is angiogenic neo-vessel diameter, d_c is the neo-vessel characteristic diameter, S_V surface area per unit volume for transvascular exchange, p_{lum} is intravascular blood pressure, p_{ins} is interstitial fluid pressure (IFP), π_{lum} and π_{ins} are respectively oncotic pressures of the intravascular plasma and interstitial fluid, κ_i is permeability coefficient of the neo-vessel wall, L_p is the hydraulic conductivity of the neo-vessel wall, which is defined in Eq.30.

P_i defined in Eq.4 is transvascular Peclet number to present the ratio of convection to diffusion across the neo-vessel wall, $P_i = (1-\sigma_{d,i})(U_{TFF}\, t_v)/D_{t,i}$. U_{TFF} is the transvascular fluid flow velocity (transvascular fluid flow, Q_{TFF}, divided by surface area of vessel, A_v), t_v is vessel wall thickness and $D_{t,i}$ is diffusion coefficient of species across vessel wall ($D_{t,i} = D_i$). If we replace $U_{TFF} = Q_{TFF}/A_v$ by Eq.27 and then describe the permeability coefficient of the neo-vessel wall, $\kappa_i = D_i / t_v$, the transvacular Peclet number is governed by Eq. 4.

In Eq.2, $c_{p,i}$ is plasma concentration of species that is assumed to be constant due to the ratio of venous to arterial plasma concentration of species is close to 1[14]. However, the plasma concentration of drug is modified by Eqs. 5 to model the physiological drug delivery under the influence of drug clearance and depletion. The drug clearance occurs due to blood recirculation into kidney and liver, modeled by the exponential term of Eq.5, and drug depletion occurs through transvascular exchange across angiogenic neo-vessel walls, modeled by the second term of Eq.5. In Eq. 5, $c_{p,ac0}$ and $c_{p,ag0}$ are initial concentrations of the anti-cancer and anti-angiogenic drugs, and τ_{ac} and τ_{ag} are mean lifetimes of anti-cancer and anti-angiogenic drugs, respectively.

$$\sigma_{d,i} = \left(1-\left(1-\frac{d_i}{d_p}\right)^2\right)^2 \tag{3}$$

$$P_i = (1-\sigma_{d,i})\left[\frac{L_p}{\kappa_i}(p_{lum} - p_{ins} - \sigma_v(\pi_{lum} - \pi_{ins}))\right] \tag{4}$$

$$c_{p,ac} = c_{p,ac0}\ e^{-t/\tau_{ac}} - S_i\ \Delta t$$
$$c_{p,ag} = c_{p,ag0}\ e^{-t/\tau_{ag}} - S_i\ \Delta t \tag{5}$$

The relationship between vessel wall pore sizes of normal tissue (stroma), d_p^s, and tumor tissue, d_p^t, is derived via scale-up of Kozeny-Carman relation as, $d_p^s / d_p^t = (L_p^s / L_p^t)^{1/6}$. As shown in Table A, we use a constant value for pore size of intertumoral vessel walls, and calculate the pore size of the vessel wall in normal tissue based on this relationship.

Reaction models for molecular scale species, *R_i*

Cellular respiration species- oxygen, glucose, and CO_2: Assumed to be a function of cellular vitality, the consumption of glucose and oxygen as well as the production of CO_2 by TCs are related by the stoichiometry of the cellular respiration reaction according to:

$$Cellular\ \ Respiration: C_6H_{12}O_6 + 6O_2 \rightarrow 6CO_2 + 6H_2O + ATP$$
$$R_g = \underbrace{-1/6\ \gamma_0 \upsilon}_{\substack{\text{consumption by}\\ \text{TCs}}} - \underbrace{\varepsilon_g c_g}_{\text{natural decay}}$$
$$R_{o_2} = -\underbrace{\gamma_0 \upsilon}_{\substack{\text{consumption by}\\ \text{TCs}}} - \underbrace{\varepsilon_{o_2} c_{o_2}}_{\text{natural decay}} \tag{6}$$
$$R_{co_2} = \underbrace{\gamma_0 \upsilon}_{\substack{\text{production by}\\ \text{TCs}}} - \underbrace{\varepsilon_{co_2} c_{co_2}}_{\text{natural decay}}$$

γ_0 is the maximum consumption or production rate of the cellular respiration species and υ is cellular vitality function defined in Eq.19, ε_g, ε_{o_2}, and ε_{co_2} are natural decay rates of glucose, oxygen, and CO_2 into interstitium.

VEGF and VEGFR-2: the hypoxic TCs exposed to oxygen concentration below a threshold level, $c_{o_2}^{ch}$, secrete VEGF to stimulate ECs of nearby vessels to sprout. The VEGF also affects vascular hydraulic conductivity/pore size (Eq. 28). Once ECs become associated with the tumor tissue, their VEGFR-2 receptors are expressed [19]. Binding to and un-binding from VEGFR-2 act as a sink and source for VEGF, respectively. Binding to an anti-angiogenic drug (anti-VEGF type) is another sink for VEGF. VEGF naturally decays in the interstitium. Therefore, the reaction model of VEGF coupled with VEGFR-2, oxygen, anti-angiogenic drug (anti-VEGF type), and ECM is governed by the set of equations 7 to 9:

$$R_v = \underbrace{r_{vegf}\left(1-\frac{c_{o_2}}{c_{o_2}^{ch}}\right)H_{(c_{o_2}^{ch}-c_{o_2})}}_{\substack{production\ by\ hypoxic\\ tumor\ cells}} - \underbrace{k_v^+ r_v^f c_v}_{\substack{bound\\ to\ VEGFR-2}} + \underbrace{k_v^- r_v^a}_{\substack{unbound\\ from\ VEGFR-2}} \underbrace{-k_{v-ag}\, c_{ag}\, c_v}_{\substack{bound\\ to\ anti-VEGF}} - \underbrace{\varepsilon_v c_v}_{natural\ decay} \tag{7a}$$

$$R_v^b = \underbrace{k_v^+ r_v^f c_v}_{\substack{bound\\ to\ VEGFR-2}} - \underbrace{k_v^- r_v^a}_{\substack{unbound\\ from\ VEGFR-2}} \tag{7b}$$

$$\frac{dr_v^f}{dt} = -k_v^+ r_v^f c_v + k_v^- r_v^a \tag{8}$$

$$\frac{dr_v^a}{dt} = k_v^+ r_v^f c_v - k_v^- r_v^a \tag{9}$$

R_v and R_v^b are the reaction rates of free VEGF and VEGF bound to VEGFR-2, respectively; c_v and c_v^b are concentrations of free and bound VEGF, respectively. r_{vegf} is the rate of production of VEGF by TCs; k_v^+ is the binding rate of VEGF to VEGFR-2; k_v^- is the dissociation rate of VEGF from VEGFR-2; k_{v-ag} is the VEGF binding rate to anti-VEGF; ε_v is the natural decay rate of VEGF; r_v^f is the concentration of free-VEGFR-2, and r_v^a is the concentration of active-VEGFR-

2 bound to VEGF. As such, $H_{(c_{o_2}^{ch}-c_{o_2})}$ is a Heaviside function to activate VEFG secretion when oxygen concentration, c_{o_2}, falls below the characteristic value, $c_{o_2}^{ch}$.

ang-1 and ang-2 and their common receptor, Tie-2: ang-1 is secreted by ECs, and ang-2 is secreted by both ECs associated with tumor tissue and hypoxic tumor cells [19-21]. There is a competition between ang-1 and ang-2 to bind to their common Tie-2 receptor. Thus, competitive binding and unbinding of Tie-2 depletes or produces, respectively, ang-1 and ang-2. The reaction models of ang-1 and ang-2 coupled with Tie-2 also include natural decay, and are mathematically modeled through the set of equations 10 to14.

$$R_{a1} = \underbrace{r_{ang-1}\left(\frac{e_0 K_a - (c_{a1})^2}{K_a}\right)}_{\text{production by endothelial cells}} - \underbrace{k_{a1}^{+}\, r_a^{f}\, c_{a1}}_{\substack{\text{bound}\\ \text{to Tie-2}}} + \underbrace{k_{a1}^{-}\, r_{a1}^{a}}_{\substack{\text{unbound}\\ \text{from active Tie-2}}} - \underbrace{\varepsilon_{a1}\, c_{a1}}_{\text{natural decay}} \tag{10a}$$

$$R_{a1}^{b} = \underbrace{k_{a1}^{+}\, r_a^{f}\, c_{a1}}_{\substack{\text{bound}\\ \text{to Tie-2}}} - \underbrace{k_{a1}^{-}\, r_{a1}^{a}}_{\substack{\text{unbound}\\ \text{from active Tie-2}}} \tag{10b}$$

$$R_{a2} = \underbrace{r_{ang-2}^{v}\left(\frac{e_0 K_a - (c_{a2})^2}{K_a}\right)}_{\substack{\text{production by}\\ \text{tumor-induced vascular remodeling}}} + \underbrace{r_{ang-2}^{h}\left(\frac{h_0 K_a - (c_{a2})^2}{K_a}\right)}_{\substack{\text{production by}\\ \text{hypoxic tumor cells}}}$$
$$- \underbrace{k_{a2}^{+}\, r_a^{f}\, c_{a2}}_{\substack{\text{bound}\\ \text{to Tie-2}}} + \underbrace{k_{a2}^{-}\, r_{a2}^{a}}_{\substack{\text{unbound}\\ \text{from active Tie-2}}} - \underbrace{\varepsilon_{a2}\, c_{a2}}_{\text{natural decay}} \tag{11a}$$

$$R_{a2}^{b} = \underbrace{k_{a2}^{+}\, r_a^{f}\, c_{a2}}_{\substack{\text{bound}\\ \text{to Tie-2}}} - \underbrace{k_{a2}^{-}\, r_{a2}^{a}}_{\substack{\text{unbound}\\ \text{from active Tie-2}}} \tag{11b}$$

$$\frac{dr_a^{f}}{dt} = -k_{a1}^{+}\, r_a^{f}\, c_{a1} + k_{a1}^{-}\, r_{a1}^{a} - k_{a2}^{+}\, r_a^{f}\, c_{a2} + k_{a2}^{-}\, r_{a2}^{a} \tag{12}$$

$$\frac{dr_{a1}^{a}}{dt} = k_{a1}^{+}\, r_a^{f}\, c_{a1} - k_{a1}^{-}\, r_{a1}^{a} \tag{13}$$

$$\frac{dr_{a2}^{a}}{dt}=k_{a2}^{+}\ r_{a}^{f}\ c_{a2}-k_{a2}^{-}\ r_{a2}^{a} \tag{14}$$

R_{a1}, R_{a2}, R_{a1}^{b}, and R_{a2}^{b} are ,respectively, reaction rates of free ang-1 and ang-2, and bound ang-1 and ang-2 to Tie-2. c_{a1}, c_{a2}, c_{a1}^{b}, and c_{a2}^{b} are, respectively, concentrations of free ang-1 and ang-2, and bound ang-1 and ang-2 to Tie-2. r_{ang-1}, r_{ang-2}^{v}, and r_{ang-2}^{h} are respectively the secretion rates of ang-1 by ECs, secretion rate of ang-2 by ECs associated with tumor tissue, and secretion rate of ang-2 by hypoxic TCs. e_0 and h_0 are respectively the characteristic concentration of ECs in each blood vessel, and the characteristic concentration of TCs, K_a is the carrying capacity coefficient of angiopoietins, k_{a1}^{+} and k_{a1}^{-} are respectively ang-1 binding rate to and unbinding rate from Tie-2, k_{a2}^{+} and k_{a2}^{-} are respectively ang-2 binding rate to and unbinding rate from Tie-2, ε_{a1} and ε_{a2} are the natural decay rates of ang-1 and ang-2, respectively. r_{a}^{f} is the concentration of free Tie-2, r_{a1}^{a} and r_{a2}^{a} are concentrations of active Tie-2 bound to ang-1 and ang-2, respectively.

To consider fibronectin concentration in the ECM coupled with matrix metalloproteins (MMPs) secreted by ECs and TCs, we applied the reaction terms of our previous model [13].

Anti-cancer and anti-angiogenic chemotherapy drugs: the anti-cancer drug is consumed by TCs and ECs and has cytotoxic effects on both cell types. In this work, the anti-angiogenic drug blocks VEGF rather than its receptor(s); its concentration decreases as it binds irreversibly to VEGF.

The drugs are assumed to naturally decay in the interstitium. The reaction models of anti-cancer and anti-angiogenic drugs are presented in Eqs. 17 and 18, respectively:

$$R_{ac} = -\underbrace{k_{ac}^{TC}\,\upsilon\,c_{ac}}_{\substack{Uptake\\ by\ tumor\ cells}} - \underbrace{k_{ac}^{EC}\,c_{ac}}_{\substack{Uptake\\ by\ endothelial\ cells}} - \underbrace{\varepsilon_{ac}c_{ac}}_{natural\ decay} \tag{17}$$

$$R_{ag} = -\underbrace{k_{ag-v}\,c_v\,c_{ag}}_{\substack{bound\\ to\ VEGF}} - \underbrace{\varepsilon_{ag}c_{ag}}_{natural\ decay} \tag{18}$$

k_{ac}^{TC} and k_{ac}^{EC} are uptake rates of anti-cancer drug by TCs and ECs, respectively, k_{ag-v} is anti-VEGF binding rate to VEGF, ε_{ac} and ε_{ag} are the natural decay rates of anti-cancer and anti-VEGF drugs, respectively.

2 Cellular Scale

Tumor cells: Similar to our previous TME model, we implement a modified cellular vitality (υ)/cellular energy (ψ) model to consider the effects of oxygen, glucose and CO_2 on TC phenotypes [13]. Cellular vitality is increased with oxygen and glucose and decreased with CO_2. Cellular energy representing available units of ATP determine bioactivity of TCs [13, 22]. The mathematical model of coupled cellular vitality and cellular energy is presented in Eqs. 19 and 20:

$$\upsilon = \varphi\left(\frac{c_{o_2}}{c_{o_2}+c_{o_2}^{ch}}+k_W\right).\ \frac{c_g}{c_g+c_g^{ch}}\ \exp\left(-5\left(\frac{c_{co2}}{c_{co2}^{ch}}-1\right)^4 H_{(c_{co_2}-c_{co_2}^{ch})}\right) \tag{19}$$

$$\frac{d\psi}{dt} = \left(k_a^p\ \upsilon - k_a^c\ \frac{\upsilon}{\upsilon+1} - k_{ac}\,c_{ac}\frac{\upsilon}{\upsilon+1}\right)H_{(\upsilon-\upsilon^{ch})} - k_q^c\ \frac{\upsilon}{\upsilon+1}H_{(\upsilon^{ch}-\upsilon)} \tag{20}$$

In Eq.19, φ is a proportionality coefficient, $c_{o_2}^{ch}$, c_g^{ch}, and c_{co2}^{ch} are oxygen, glucose, and carbon dioxide characteristic concentrations, respectively [13, 23]. $H_{(c_{co_2}-c_{co_2}^{ch})}$ is a Heaviside function to

ensure that CO_2 reduces cellular vitality when it's concentration, c_{co_2}, exceeds the characteristic value, $c_{co_2}^{ch}$. k_W is a constant to reproduce the Warburg effect of TCs, which tends to favor metabolism via glycolysis rather than the oxidative phosphorylation, which is the preference of most other cells in the body. Therefore, if the oxygen concentration of TCs approaches zero, the TCs can survive but with very low vitality.

In this model, the TCs with υ below υ^{ch} are assumed to be quiescent and those with υ above υ^{ch} are active [13]. The active TCs need to achieve a characteristic energy, ψ^{ch}, before they can proliferate into two new TCs [13, 24, 25].

The active TCs produce ATP at a linear rate related to cellular vitality with a proportional coefficient, k_a^p, and also consume cellular energy based on a M-M model with maximum rate k_a^c and M-M constant 1 [9, 13]. The quiescent TCs consume ATP according to a M-M model with maximum rate k_q^c and M-M constant 1. Quiescent TCs with zero cellular energy are converted to necrotic phenotype. Indeed, quiescent TCs can be converted to an active or necrotic state based on cellular vitality and energy, and active TCs can become quiescent; however, necrotic TCs cannot be converted to the other phenotypes. In this model, the anti-cancer drug is assumed to interfere with DNA repair, thereby reducing cellular energy of the active TCs. According to Eq. 20, the effect of anti-cancer drug on TCs is imposed with a M-M model with a drug-dependent maximum rate, $k_{ac}\, c_{ac}$, and M-M constant equal to 1.

Endothelial cells: During angiogenesis, endothelial tip cells (tECs) migrate toward positive gradients of VEGF [13, 26-28] and stalk endothelial cells (sECs) migrate into the tECs-generated conduits in the ECM and also proliferate to create lumens of the angiogenic neo-vessels [13, 26,

29, 30]. The death state is also considered for sECs based on VEGF concentration (see Eq.23). Moreover, the sECs can differentiate into tECs in response to high VEGF concentration and high ratio of ang-2 to ang-1, and thus generate bifurcating branches from the neo-vessel wall [26, 31]. The branching probability function is presented in Eq. 21.

$$P_{Br} = m_{Br} \frac{c_v^b}{c_v^b + k_{Br,v}} \frac{(c_{a2}^b / (c_{a1}^b + s_{Br,a}))}{(c_{a2}^b / (c_{a1}^b + s_{Br,a})) + k_{Br,a}} \tag{21}$$

m_{Br} is the maximum probability of branching, $k_{Br,v}$, and $k_{Br,a}$ are positive constants to control the impacts of VEGF and the ang-2 / ang-1 ratio on branching; $s_{Br,a}$ is a positive constant to avoid the singularity due to zero concentration of ang-1.

3.1 Tissue scale: development of tumor tissue and neo-vessel pathways

In response to high VEGF concentration and VEGF gradients, tECs migrate into the ECM to create pathways for angiogenic neo-vessels. Tumor cells can sense the oxygen- and nutrient-rich regions in the tissue as well as cell density in the surrounding tissue. In addition to biochemical agents, tumor-induced solid pressure presents a resistance to the migration of TCs and tECs [9, 13, 32-34]. New TCs are stimulated to migrate toward the locations with high oxygen and nutrients (which can result in cooption of tumor vessels), low solid pressure (i.e., low viable cell concentration). We assume that newly-divided TCs can displace ("crowd") viable cells, but not necrotic cells. tECs are motivated to migrate toward high VEGF concentration regions and low solid pressure. The tECs cannot penetrate the regions occupied by necrotic TCs. The fibronectin gradient in the ECM caused by TC- and tEC-induced MMPs supports haptotactic migration of TCs and tECs [13].

3.2 Tissue scale: Vessel growth and remodeling

Lumenogenesis and vessel adaptation with growth factor and shear stress: After new vessels form via angiogenesis, they need to form lumens before flow can proceed. They do this through a process of lumenogenesis, which is controlled by VEGF, ang-1, and ang-2. The equation for neo-vessel diameter, d_v, is written as Eq. 22,

$$d_v = \frac{G_s}{G_s + G_0} d_c \tag{22}$$

G_s is the growth function of angiogenic neo-vessels and G_0 is a M-M constant. G_s depends on the proliferation and death rates of sECs as well as their WSS-induced mechanotransduction, according to [35, 36]:

$$\frac{dG_s}{dt} = \underbrace{\alpha_p \left(\frac{c_v^b}{c_v^b + \theta_p} \right) \underbrace{\left(1 - \delta_{ac} \frac{c_{ac}}{c_{ac} + \theta_{ac}} \right)}_{anti-cancer\ sEC\ \text{cytotoxicity}}}_{sEC\ proliferation} - \underbrace{\delta \left(1 - \frac{c_v^b}{c_v^b + \theta_d} \right)}_{sEC\ death} + \underbrace{k_\tau (\tau_{WSS} - \tau_{WSS,ref}) H_{(c_v - c_{v,\min})} H_{(c_{v,\max} - c_v)}}_{Mechanotransduction-induced\ lumen\ growth} \tag{23}$$

α_p and θ_p are, respectively, the maximum rate and the M-M constant for sEC proliferation; δ_{ac} and θ_{ac} are, respectively, the maximum rate and M-M constant for anti-cancer sEC cytotoxicity; δ and θ_d are the maximum rate and M-M constant for sEC death, respectively. k_τ is also a positive constant that represents the neo-vessel lumen growth due to wall shear stress (WSS), $\tau_{WSS,ref}$ is a reference value for WSS, $c_{v,\min}$ and $c_{v,\max}$ are threshold concentrations of VEGF, between which the WSS effect dominates.

Vessel deformation and adaptation with pressure: the angiogenic neo-vessels, which have a constant elasticity, E, and compliance power, cp, as well as a collapse pressure, p_c, are deformed through Eq. 24 [13, 37-39].

$$d_{v,def} = d_v\left(\frac{p_{lum} - (p_{ins} + p_s) + p_c}{E}\right)^{cp} \tag{24}$$

p_{lum}, p_{ins}, and p_s are, respectively, intravascular pressure, IFP, and tumor growth-induced solid stress; d_v is vessel diameter and $d_{v,def}$ is deformed vessel diameter.

3.3 Tissue scale: Fluid dynamics of TME

Fluid transport is explicitly considered in the model. This includes The TME flow within vascular lumens, transvascular fluid flow, and interstitial fluid flow (IFF). Intravascular blood flow, governed by Hagen-Poiseuille's law, and IFF, calculated using Darcy's, law are coupled with each other through transvascular fluid flow calculated by Starling's law [6, 11, 13, 40]. Equation 25 shows the continuity of intravascular blood flow. In this equation, Q_{lum} is the blood flow rate in the lumen calculated as the difference between intravascular blood flow rate, Q_{IBF}, based on Hagen-Poiseuille's law, Eq. 26, and transvascular fluid flow rate, Q_{TFF}, based on Starling's law, Eq. 27.

$$\sum_{b=1}^{N} Q_{lum}^{b} \beta^{b} = 0 \quad , \quad Q_{lum} = Q_{IBF} - Q_{TFF} \tag{25}$$

$$Q_{IBF} = \frac{\pi}{128} \frac{\Delta p_{lum} \; d_v^{\,4}}{L \; \mu_{blood}(d_v, H_D)} \tag{26}$$

$$Q_{TFF} = (\pi d_v \, L) L_p (p_{lum} - p_{ins} - \sigma(\pi_{lum} - \pi_{ins})) \tag{27}$$

In Eq. 25, N is the number of peripheral vessel lattice nodes adjacent to the central vessel node, and β describes direction of lumen blood flow (+1 for outlet flow from a peripheral node and -1 for inlet flow to a peripheral node). In Eq. 26, L is the length of a neo-vessel segment, and μ_{blood} is

the dynamic non-Newtonian viscosity of blood as a function of neo-vessel diameter, d_v, and blood hematocrit, H_D, calculated using our previous hemorheology model [13]. In Eq. 27, L_p is the hydraulic conductivity of the neo-vessel wall, which is defined in Eq.28 as a function of VEGF and the ratio of ang-2 to ang-1 [38, 41, 42].

$$L_p = L_p^0 \left(1 + k_p \frac{c_v^b}{c_v^b + k_{p,v}} \left(1 + \frac{(c_{a2}^b / c_{a1}^b)}{(c_{a2}^b / c_{a1}^b) + k_{p,a}} \right) \right) \tag{28}$$

k_p is a positive constant to limit the increment of L_p in response to VEGF and ang-2/ang-1, $k_{p,v}$ and $k_{p,a}$ are positive constants to control the effect of VEGF and ang-2/ang-1 on L_p, L_p^0 is a reference value for vessel wall hydraulic conductivity. The continuity equation for IFF given in Eq. 29 shows the incompressibility of plasma in avascular tissue and leakiness of the neo-vessel wall in vascular tissue. Darcy's law, which determines IFF, is also shown in Eq. 30.

$$\nabla . u_{ins} = \begin{cases} \dfrac{Q_{TFF}}{V} = L_p \, S_V \left((p_{lum} - p_{ins} - \sigma(\pi_{lum} - \pi_{ins})) \right) & \textit{Vascular tisuue} \\ 0 & \textit{Avascular tisuue} \end{cases} \tag{29}$$

$$u_{ins} = -K_{ins} \nabla p_{ins} \tag{30}$$

By combining Eq.30 and Eq. 29, the Poison-Laplace's equation for IFP is derived (Eq. 31):

$$-\nabla^2 p_{ins} = \begin{cases} \dfrac{L_p}{K_{ins}} S_V \left((p_{lum} - p_{ins} - \sigma(\pi_{lum} - \pi_{ins})) \right) & \textit{Vascular tissue} \\ 0 & \textit{Avascular tissue} \end{cases} \tag{31}$$

$$K_{ins} = \begin{cases} K_{ins}^t \left(1 - k_{ps} \dfrac{p_s}{p_s + p_0} \right) & \textit{Tumor tissue} \\ K_{ins}^s & \textit{Stroma tissue} \end{cases} \tag{32}$$

K_{ins} is the interstitial hydraulic conductivity of the TME defined as a function of tumor-induced solid pressure in Eq.32, [6, 7]. K_{ins}^{t} and K_{ins}^{s} are respectively the interstitial hydraulic conductivity of tumor and normal stroma tissue, k_{ps} is coefficient describing the reduction of hydraulic conductivity due to tumor fibrosis, and p_0 is a characteristic TME pressure.

Accumulation of rapidly dividing TCs increases the mechanical, compressive solid stress, p_s [9, 43]. For this additional tumor growth-induced stress, we chose the Gaussian-like function for accumulative systems [9, 13]. The model parameters are presented in Table A.

3.4 Initial and boundary conditions

The initial concentrations of glucose, oxygen, and carbon-dioxide were assumed to be homogeneous. The initial concentrations of VEGF, ang-1, and ang-2 are set to zero. The free and active VEGFR-2 and Tie-2 are initialized to zero. At the boundaries of the computational domain of the TME, a Dirichlet boundary condition was used for each agent with value equal to its initial concentration. For drug boundary condition, we assume the same equation as drug plasma concentration to consider the effect of far field primary vessels. As in our previous model [13], the TME was seeded with five tumor cells located at the center of the computational cube and a hypothetical circular primary vascular network with a radius approximately 5 mm. The locations of initial sprouts on the circle of primary vessels are determined based on VEGF concentration but spaced randomly according to NOTCH induction more than 50 μm apart.

The biomechanical factors, including IFF velocity and IFP, intravascular blood flow velocity and pressure, and WSS were initially set to zero in the entire computational domain and boundaries.

For these parameters, a Dirichlet boundary condition with zero value was set on all boundaries of the TME domain. To allow for increased blood supply and vascular maturation with tumor growth, we assume the surrounding supply vessels grow and their pressure increase as the tumor grows: at the inlet of the neo-vessels where they connect to the primary vessel, we developed a M-M model to calculate inlet pressure as a function of tumor size; $p_{inlet} = p_m (V_T / (V_T^m + V_T))$, where V_T is tumor volume, V_T^m is M-M constant, and p_m is the maximum pressure in the primary vessel, consistent with the range reported in the literature [7, 10, 44].

Assumptions and limitations: A) the primary vessels are assumed to be on a circular region. B) drug concentration of far-field is assumed to be negligible. C) The dynamics of stromal cells are not considered.

The effect of area under the cure (AUC) of anti-cancer drug on tumor expansion

The area under the curve (AUC) of the drug administration plot is a determinant of the performance of chemotherapy. In Figure A, we show the effects of metronomic therapy when it has the same AUC as MTD compared to higher AUC than MTD. According to these results, M with the same AUT as MTD achieves better therapeutic results compared to MTD, which would even be improved by increasing AUC of M (viable tumor cells in Figure A-i). In terms of drug accumulation in tumor and normal tissue shown in Figures A- ii and iii, respectively, decreasing the AUC of M significantly decreases accumulation of drug in the tumor, but slightly decreases the accumulation of drug in normal tissue.

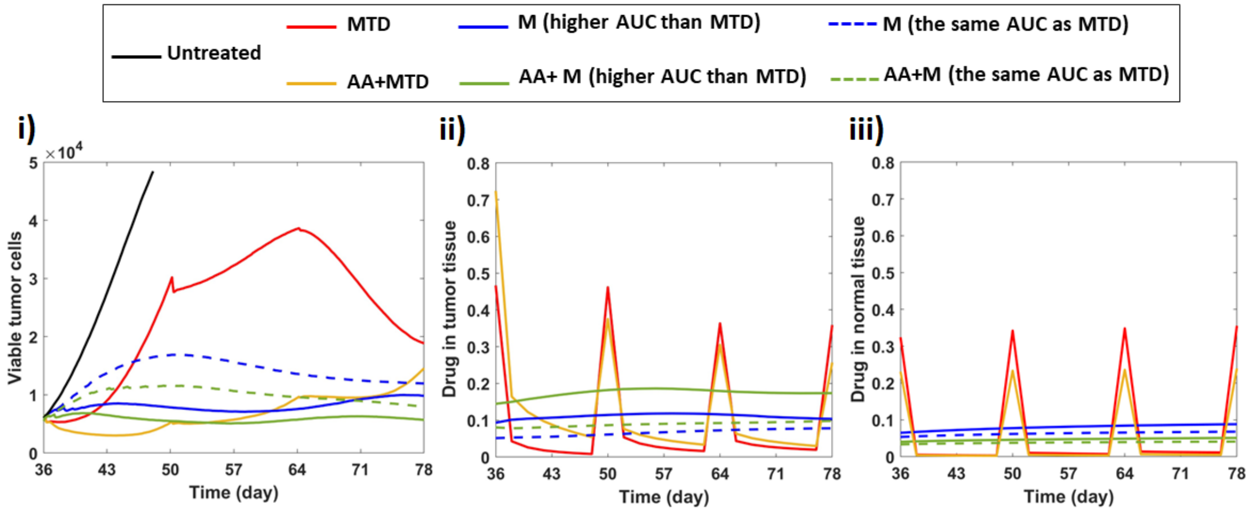

Figure A. Effect of area under the curve (AUC) of anti-cancer drug on cancer killing and toxicity, A) viable cancer cell number (proliferating plus quiescent). Drug concentration in the tumor (B) and normal tissue (C) during different chemotherapy regimens (MTD anti-cancer "MTD," metronomic anti-cancer "M", combination anti-angiogenesis and MTD treatment "AA+MTD", and combination anti-angiogenic and metronomic, "AA+M"). the drug concentrations are normalized with the MTD injection dose.

The effect of different treatments on tumor expansion morphology

To quantitatively compare the invasion of tumor cells in the x-y plane and in the +/- z directions, we calculated the locations of tumor cells in the x-y plane and in the +/- z directions to determine the maximum progression of cancer in the +/- z directions and in the x-y plane shown in Figures B-i and ii, respectively. The results show that metronomic therapy compared to MTD can enhance tumor expansion in the z direction against the angiogenic vessels. Combination therapy of MTD treatment and ant-VEGF can decrease maximum tumor expansion in both z and x-y directions, but combining anti-VEGF with metronomic treatment can increase maximum tumor expansion in all directions. The MTD treatment and the combination therapy with MTD and anti-VEGF result in a

flattened tumor with very low z/x-y aspect ratio compared to the metronomic treatment and metronomic combined with anti-VEGF (Figure B-iii).

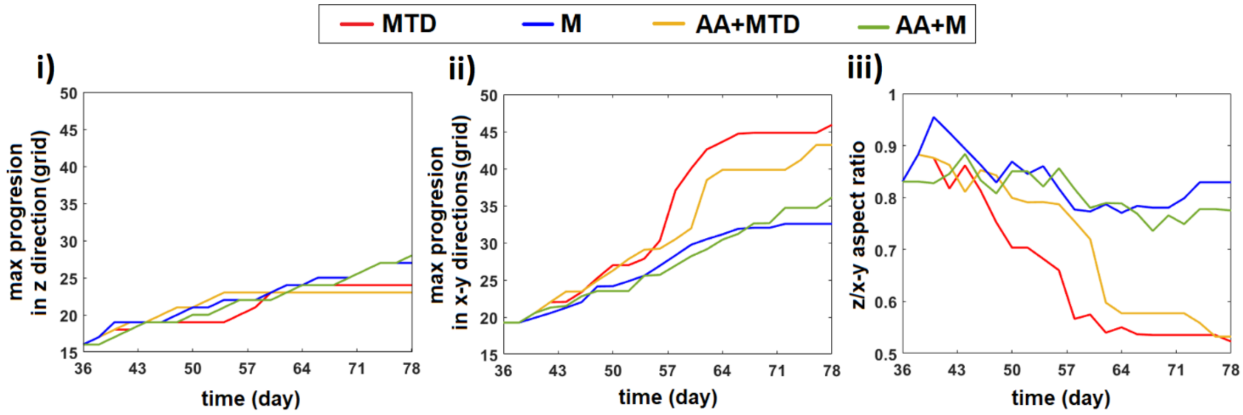

Figure B. Analysis of tumor expansion patterns, i) maximum expansion of tumor cells in the +/- z direction, ii) maximum expansion of tumor cells in the x-y plane, iii) z/xy aspect ratio of tumor.

Validation of model predictions with experimental observations

Our simulated results compare well with experimental results and other computational models. We predict tumor size reduction measured by Yapp, Wong (45) for metronomic Gemcitabine therapy (treatment duration= 7 days; Figure C- i).

We also compared our model results for intratumoral IFP during anti-VEGF treatment with experimental data of Turley, Fontanella (46), Figure C- ii. The results show an IFP reduction around ~50% after treatment with anti-VEGF (Bevacizumab).

We predict the tumor size reduction in response to anti-cancer drug (doxorubicin, 20 µg/ml administered once two days) that reproduces the experimental measurements of Sengupta, Eavarone (47) (Figure C- iii). Our model also agrees with the anti-angiogenic-induced reduction of tumor vessel density measured by Zhou, Zhang (48) (treatment duration= 7 days; Figure C- iv).

The results for IFP and IFV are compared with the results of Jain, Tong (2), Figure C- v. The current results are in good agreement with the Jain, et al. model, showing that IFP is uniformly high within the tumor but rapidly decreases in the normal surrounding stroma. Both models also show that IFV is maximum at the tumor boundary and decreases within the tumor and normal stroma, Figure C- v.

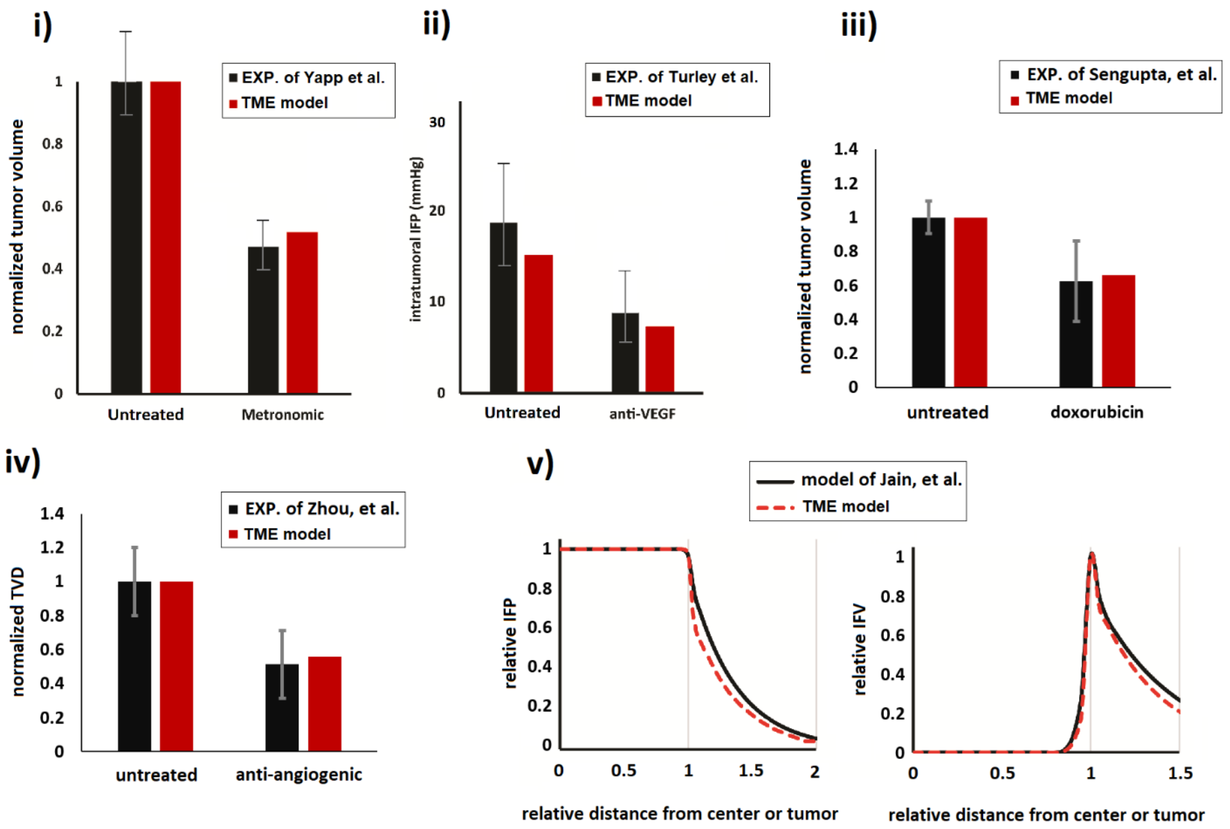

Figure C. Model validation. Comparison of the model predictions with experimental measurements, i) normalized tumor size for untreated and metronomic q3d of gemcitabine (low mean lifetime drug) for 7 days. ii) intratumoral IFP for untreated and anti-VEGF treated tumor. iii) normalized tumor size for untreated and doxorubicin treatment for 7 days, iv) normalized tumor vessel density (TVD) for untreated and anti-angiogenic treatment, v) relative IFP from the center of tumor to stroma, 1 (in vertical axis) shows the tumor-stroma interface (left), relative interstitial fluid velocity (IFV) from the center of tumor to stroma, 1 (in vertical axis) shows the tumor-stroma interface (right).

Parameter sensitivity analysis

We performed a parameter sensitivity analysis to determine the effect of key parameters on the model outcomes, including drug accumulation in the tumor (averaged value of drug concentration in tumor tissue) and number of viable tumor cells. The considered model parameters are the reference values for vessel wall hydraulic conductivity, L_p^0, surface area per unit volume for transvascular exchange, S_V, intravascular blood pressure, [illegible] that is controlled by maximum

pressure in primary vessels (p_m), initial drug concentration (IDC), and frequency of drug administration (FDA). The control case we use for the baseline of the parametric study is the tumor treated with metronomic therapy administered daily (20% MTD). In this study, L_p^0, S_V, p_{lum}, IDC, and FDA parameters were increased by 20% (indicated with ↑) and 40% (indicated with ↑↑) of the baseline values (see Table A); we also decreased the values by 20% (indicated with ↓) and 40% (indicated with ↓↓).

The results of the parameter sensitivity analysis simulated for 78 days are shown in Figure D. Figure D- i shows the sensitivity of drug accumulation in the tumor to variations of the parameters L_p^0, S_V, p_{lum}, IDC, and FDA. These results show that drug accumulation in the tumor has an inverse correlation with L_p^0, S_V, p_{lum} and a direct correlation with IDC and FDA. Drug accumulation in the tumor is most sensitive to FDA and then IDC. Figure D- ii shows the sensitivity of viable tumor cells to the variations in L_p^0, S_V, p_{lum}, IDC, and FDA. The number of viable tumor cells has a direct correlation with L_p^0, S_V, p_{lum} and an inverse correlation with IDC and FDA. The number of viable tumor cells is most sensitive to FDA.

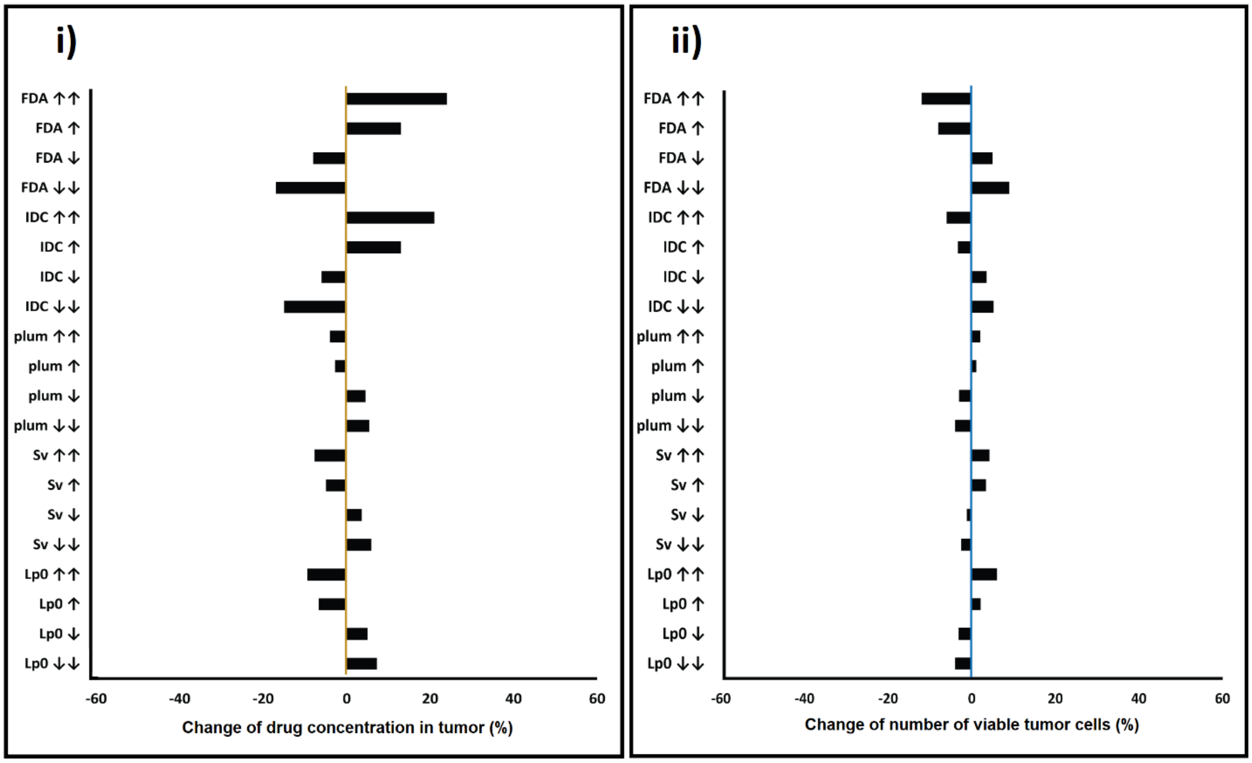

Figure D. Parameter sensitivity analysis for the model by predicting the change of main outcomes in response to varying model parameters. The model outcomes include drug accumulation in tumor (i) and number of viable tumor cells (ii). The symbols ↑ and ↑↑ indicate the increment of parameter values by 20% and 40% of the baseline value, and the symbols ↓ and ↓↓ indicate reduction of parameter value by 20% and 40% of the baseline value.

Anti-cancer drug pharmacokinetics affect metronomic therapy

In this study, we focused on a single anti-cancer drug (Cisplatin). Because the drug circulation and clearance times are critical determinants of delivery and accumulation, drug pharmacokinetics must be considered carefully when developing metronomic or combination therapies. In general, anti-cancer drugs with shorter half-lives are more amenable to metronomic therapy, because extended periods between doses allows the tumor to recover. Indeed, comparing Cisplatin (plasma half-life = 30 min) with a putative drug with longer half-life (3 hr) shows less difference between MTD and metronomic scheduling (Figure E). However, even for drugs with slower clearance,

metronomic therapy may have advantages in terms of lower toxicity, as the systemic plasma distribution does not experience the large spikes associated with MTD administration.

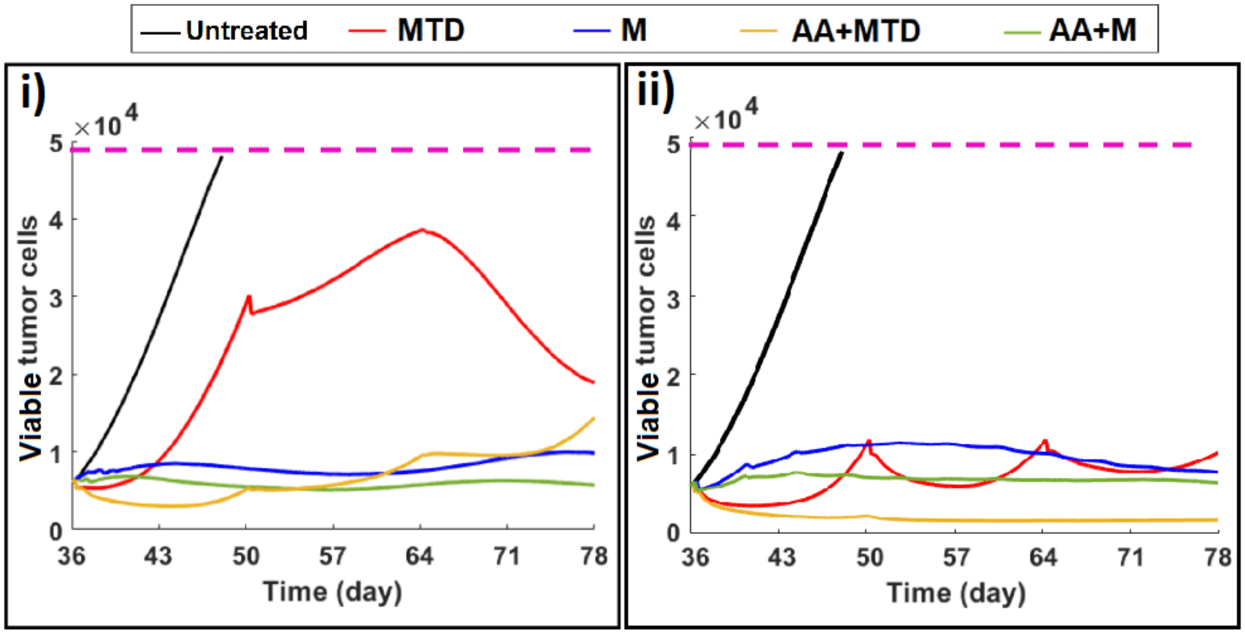

Figure E. Effect of anti-cancer drug half-life on responses to single and combination therapies. i) viable tumor cells treated with cisplatin (half-life of 30 min). ii) viable tumor cells treated with a putative anti-cancer drug with longer half-life (3 hr).

Model Parameters

Table A. The parameters used for computational results of the mathematical model.

Parameters	Description	Value	References
Cellular respiration			
D_{o_2}	Diffusion coefficient of oxygen	1×10^{-9} m^2/s	Anderson (49)
D_g	Diffusion coefficient of glucose	3.6×10^{-10} m^2/s	Jain (50)
D_{co_2}	Diffusion coefficient of carbon-dioxide	7.4×10^{-10} m^2/s	Laursen and Kirk (51)
γ_0	Maximum consumption/production rate of CR-agents	3×10^{-8} M/s	Tang, Van De Ven (9)
$c_{o_2}^{ch}$	Characteristic oxygen concentration (threshold value for hypoxia)	8.4×10^{-3} M	Tang, Van De Ven (9)
c_g^{ch}	Characteristic glucose concentration (threshold value for hypoglycemia)	5.5×10^{-3} M	Bao, Chen (52)
$c_{co_2}^{ch}$	Characteristic carbon-dioxide concentration	10.5×10^{-3} M	Tang, Van De Ven (9)
c_{p,o_2}	plasma concentration of oxygen	8.4×10^{-3} M	Tang, Van De Ven (9)
$c_{p,G}$	plasma concentration of glucose	5.5×10^{-3} M	Bao, Chen (52)
c_{p,co_2}	plasma concentration of CO2	1.2×10^{-3} M	Higgins (53)
ε_{o2}	Natural decay rate of oxygen	1×10^{-9} 1/s	Estimated
ε_g	Natural decay rate of glucose	1.7×10^{-10} 1/s	Estimated
ε_{co2}	Natural decay rate of carbon-dioxide	1×10^{-10} 1/s	Estimated
r_f	retardation factor	1	Baxter and Jain (14)
Growth factors			
D_v	Diffusion coefficient of VEGF	2.9×10^{-11} m^2/s	Anderson and Chaplain (54)
r_{vegf}	VEGF production rate by TCs	2×10^{-12} M/s	Tang, Van De Ven (9)
k_v^+	Binding rate of VEGF by VEGFR-2	1.3×10^{-2} 1/(μM.s)	Baldwin, Catimel (55)
k_v^-	Unbinding rate of VEGF from VEGFR-2	6.3×10^{-5} 1/s	Baldwin, Catimel (55)
ε_v	Natural decay rate of VEGF	2.78×10^{-7} 1/s	Gevertz and Torquato (19)
c_v^{ch}	Characteristic VEGF concentration	1.1×10^{-8} M	Tang, Van De Ven (9)
$c_{p,v}$	plasma concentration of VEGF	5.78 pg/ml	McIlhenny, George (56)
Angiopoietins			
D_{a1}	Diffusion coefficient of ang-1	Very small →0 m^2/s	Gevertz and Torquato (19)
D_{a2}	Diffusion coefficient of ang-2	12.5×10^{-13} m^2/s	Billy, Ribba (57)
r_{ang-1}	Secretion rate of ang-1 by ECs	2.78×10^{-6} 1/s	Gevertz and Torquato (19)
r_{ang-2}^v	Secretion rate of ang-2 by ECs associated with tumor tissue	2.22×10^{-5} 1/s	Gevertz and Torquato (19)

Parameters	Description	Value	References
r_{ang-2}^{h}	secretion rate of ang-2 by TCs	1.4×10^{-5} 1/s	Gevertz and Torquato (19)
k_{a1}^{+}	Binding rate of ang-1 to Tie-2	1×10^{-2} 1/(μM.s)	Davis, Aldrich (58)<br>Longstaff (59)
k_{a1}^{-}	Unbinding rate of ang-1 from Tie-2	3.7×10^{-5} 1/s	Davis, Aldrich (58)<br>Longstaff (59)
k_{a2}^{+}	Binding rate of ang-2 to Tie-2	1.16×10^{-2} 1/(μM.s)	Maisonpierre, Suri (60)<br>Gevertz and Torquato (19)
k_{a2}^{-}	Unbinding rate of ang-2 from Tie-2	3×10^{-5} 1/s	Maisonpierre, Suri (60)<br>Gevertz and Torquato (19)
K_a	Carrying capacity coefficient of angiopoietins	1.5×10^{-2} μM	Gevertz and Torquato (19)
e_0	Characteristic concentration of ECs at each blood vessel	1×10^{-4} μM	Plank, Sleeman (61)
h_0	Characteristic concentration of TCs	1×10^{-3} μM	Gevertz and Torquato (19)
ε_{a1}	Natural decay rates of ang-1	8.33×10^{-7} 1/s	Gevertz and Torquato (19)
ε_{a2}	Natural decay rates of ang-2	5.56×10^{-7} 1/s	Gevertz and Torquato (19)
$c_{p,a1}$	plasma concentration of ang-1	6×10^{3} pg/ml	Engin, Üstündağ (62)
$c_{p,a2}$	plasma concentration of ang-2	2×10^{3} pg/ml	Engin, Üstündağ (62)
Extracellular matrix			
D_m	Diffusion coefficient of MMPs	1×10^{-13} m²/s	Cai, Xu (63)
$r_{m,T}$	Secretion rates of MMPs by TCs	1.7×10^{-13} M/s	Nikmaneshi, Firoozabadi (13)
$r_{m,E}$	Secretion rates of MMPs by ECs	0.3×10^{-13} M/s	Nikmaneshi, Firoozabadi (13)
ε_m	Natural decay rates of MMPs	1.7×10^{-8} 1/s	Cai, Xu (63)
ε_e	Natural decay rates of ECM	1.3×10^{-7} 1/s	Cai, Xu (63)
c_e^{ch}	Characteristic ECM concentration	1.36×10^{-9} M	Nikmaneshi, Firoozabadi (13)
c_m^{ch}	Characteristic MMP concentration	1.36×10^{-9} M	Nikmaneshi, Firoozabadi (13)
$c_{p,m}$	plasma concentration of MMP	72 ng/ml	Masuhara, Nakai (64)
Cellular vitality and energy			
φ	Proportionality coefficient of consumption/production rate of CR-agents	3.67	Buchwald (23)
υ^{ch}	Characteristic cellular vitality for active TCs	0.5	Nikmaneshi, Firoozabadi (13)
ψ^{ch}	Characteristic cellular energy for proliferation	30	Nikmaneshi, Firoozabadi (13)
k_q^c	Constant consumption rate of cellular energy by quiescent TCs	0.1	Tang, Van De Ven (9)<br>Nikmaneshi, Firoozabadi (13)
k_a^p	Coefficient of production rate of cellular energy by active TCs	1	Nikmaneshi, Firoozabadi (13)
k_a^c	Maximum consumption rate of cellular energy by active TCs	1	Nikmaneshi, Firoozabadi (13)
k_W	a constant to demonstrate the Warburg effect of TCs	1×10^{-10}	Estimated
Tumor growth and angiogenesis			

Parameters	Description	Value	References
α	1	Saturation coefficient of chemotaxis	Cai, Xu (63)
β_c	0.26 $m^2/(M.s)$	Weight coefficient of chemotaxis	Cai, Xu (63)
β_h	0.1 $m^2/(M.s)$	Weight coefficient of haptotaxis	Cai, Xu (63)
β_{COP}	0.3 $m^2/(M.s)$	Weight coefficient of Cooption	Estimated
D_{tEC}	Diffusivity of tECs	1×10^{-13} m^2/s	Cai, Xu (63)
D_{TC}	Diffusivity of TCs	1×10^{-13} m^2/s	Cai, Xu (63)
Vessel growth and remodeling			
G_0	M-M constant of neo-vessel lumen growth	500	Nikmaneshi, Firoozabadi (13)
α_p	Maximum rate of sEC proliferation	0.198 1/h	Cameron and Davis (35)
θ_p	M-M constant of sEC proliferation	2.8×10^{-6} mol VEGF/ 1 m^3 EC	Cameron and Davis (35)
δ	Maximum rate of sECs death	0.198 1/h	Cameron and Davis (35)
δ_{ac}	maximum rate for anti-cancer sECs cytotoxicity	0.67 1/h	Estimated, Woodley-Cook, Shin (65)
θ_{ac}	M-M constant for anti-cancer sECs cytotoxicity	5.2 µg/ml	Estimated, Woodley-Cook, Shin (65)
θ_d	M-M constant of sECs death	3.3×10^{-7} mol VEGF/ 1 m^3 EC	Cameron and Davis (35)
$\tau_{WSS,ref}$	A positive constant as reference of WSS	7.73×10^{-5} mmHg	Pries, Secomb (66)
$c_{v,\min}$	Minimum threshold concentrations of VEGF, above which the WSS effect can be dominant	1.1×10^{-11} M	Estimated
$c_{v,\max}$	Maximum threshold concentrations of VEGF, below which the WSS effect can be dominant	1.1×10^{-5} M	Estimated
k_p	Proportional coefficient of transvascular stimuli	0.5 1/s	Stéphanou, McDougall (67)
k_m	Proportional coefficient of metabolic stimuli	0.12 1/s	Stéphanou, McDougall (67)
S_{Sh}	Inherent tendency of vessels to shrink and decrease their diameter	0.35	Stéphanou, McDougall (67)
E	Constant elasticity of neo-vessels	6.5 mmHg	Netti, Roberge (37)
cp	Compliance power of neo-vessels	0.19	Netti, Roberge (37)
p_c	Collapse pressure of neo-vessels	3 mmHg	Netti, Roberge (37)
d_c	Characteristic diameter of neo-vessels	50 µm	Nikmaneshi, Firoozabadi (13)
m_{Br}	Branching constant	0.3×10^{-3}	Tang, Van De Ven (9)
$k_{Br,v}$, $k_{Br,a}$	positive constants to control the impact of ratio of ang-2 to ang-1 on branching	1	Estimated
Hemodynamics-interstitial fluid flow of TME			
K^s_{ins}	Interstitial hydraulic conductivity of TME for normal tissue	8.53×10^{-9} cm2/(mmHg.s)	Baxter and Jain (14)
K^t_{ins}	Interstitial hydraulic conductivity of TME for tumor tissue	4.13×10^{-8} cm2/(mmHg.s)	Baxter and Jain (14)
k_{ps}	tumor-induced reduction coefficient of hydraulic conductivity	1	Estimated

Parameters	Description	Value	References
L_p^0	Reference value for Hydraulic conductivity of angiogenic neo-vessels wall	3.6×10^{-10} m/(mmHg.s)	Cai, Zhang (38), Cai, Wu (42)
k_L	constant to control the effect of VEGF and ang-2/ang-1 on vessel wall hydraulic conductivity	3.39	Baxter and Jain (14)
$k_{L,v}$	constant to control the effect of VEGF on vessel wall hydraulic conductivity	1.1×10^{-8} M (equal to characteristic value of VEGF)	Estimated
$k_{L,a}$	constant to control the effect of ang-2/ang-1 on vessel wall hydraulic conductivity	1	Estimated
σ_v	average oncotic reflection coefficient of plasma proteins	0.91 for healthy tissue and 0.82 for tumor tissue	Soltani and Chen (6), Zhao, Wu (7)
π_{lum}	collide osmotic (oncotic) pressures of intravascular plasma	20 mmHg for both healthy and tumor tissues	Soltani and Chen (6), Zhao, Wu (7)
π_{ins}	collide osmotic (oncotic) pressures of interstitial fluid	10 mmHg for heathy tissue and 15 mmHg for tumor tissue	Soltani and Chen (6), Zhao, Wu (7)
S_V	Characteristic value of surface area of neo-vessels per unit volume for mass transport in the interstitium	7×10^3m^{-1} for healthy tissue and 2×10^4m^{-1} for tumor tissue	Soltani and Chen (6), Zhao, Wu (7)
p_0	Characteristic TME pressure and WSS	60 mmHg	Tang, Van De Ven (9)
p_m	the maximum pressure in the primary vessels	60 mmHg	[7, 10, 44]
d_p^t	Intratumoral vessel wall pore size	400 nm	Stylianopoulos and Jain (68)
κ_i	Permeability coefficient of vessel wall	5.73×10^{-9} cm/s for tumor tissue<br>0.73×10^{-9} cm/s for normal tissue	Baxter and Jain (15)
Anti-cancer drug and Anti-angiogenic agent			
D_{ac}	Diffusion coefficient of anti-cancer drug	2.5×10^{-13} m^2/s	Pluen, Boucher (69)
D_{ag}	Diffusion coefficient of anti-angiogenic drug	4×10^{-11} m^2/s	Yonucu, Yılmaz (1)
$k_{r_v^f-ag}$	binding rate of anti-angiogenic agent to VEGFR-2	2.6×10^4 1/(M.s)	Estimated, Baldwin, Catimel (55)
k_{ac}^{TC}	uptake rates of anti-cancer drug by TCs	2.5×10^{-7} 1/s	Estimated, Tang, Van De Ven (9)
k_{ac}^{EC}	uptake rates of anti-cancer drug by ECs	1×10^{-7} 1/s	Estimated, Tang, Van De Ven (9)
ε_{ac}	natural decay rate of anti-cancer drug	1×10^{-8} 1/s	Tang, Van De Ven (9)
ε_{ag}	natural decay rate of anti-angiogenic drug	1.65×10^{-6} 1/s	Yonucu, Yılmaz (1)
k_{ac}	proportional coefficient of anti-cancer drug activity	10	Estimated, Tang, Van De Ven (9)
τ_{ac} / 1.443	Half-life of anti-cancer drugs	30 min	Kitajima, Fukuoka (70)
τ_{ag} / 1.443	Half-life of anti-angiogenic drug	20 days	Gaudreault, Bruno (71)

References

1. Yonucu S, Yılmaz D, Phipps C, Unlu MB, Kohandel M. Quantifying the effects of antiangiogenic and chemotherapy drug combinations on drug delivery and treatment efficacy. PLoS computational biology. 2017;13(9):e1005724.
2. Jain RK, Tong RT, Munn LL. Effect of vascular normalization by antiangiogenic therapy on interstitial hypertension, peritumor edema, and lymphatic metastasis: insights from a mathematical model. Cancer research. 2007;67(6):2729-35.
3. Xu J, Vilanova G, Gomez H. A mathematical model coupling tumor growth and angiogenesis. PloS one. 2016;11(2):e0149422.
4. Voutouri C, Kirkpatrick ND, Chung E, Mpekris F, Baish JW, Munn LL, et al. Experimental and computational analyses reveal dynamics of tumor vessel cooption and optimal treatment strategies. Proceedings of the National Academy of Sciences. 2019;116(7):2662-71.
5. Kashkooli FM, Soltani M, Rezaeian M, Taatizadeh E, Hamedi M-H. Image-based spatio-temporal model of drug delivery in a heterogeneous vasculature of a solid tumor—Computational approach. Microvascular research. 2019;123:111-24.
6. Soltani M, Chen P. Numerical modeling of interstitial fluid flow coupled with blood flow through a remodeled solid tumor microvascular network. PloS one. 2013;8(6):e67025.
7. Zhao G, Wu J, Xu S, Collins M, Long Q, König CS, et al. Numerical simulation of blood flow and interstitial fluid pressure in solid tumor microcirculation based on tumor-induced angiogenesis. Acta Mechanica Sinica. 2007;23(5):477-83.
8. Norton K-A, Popel AS. Effects of endothelial cell proliferation and migration rates in a computational model of sprouting angiogenesis. Scientific reports. 2016;6:36992.
9. Tang L, Van De Ven AL, Guo D, Andasari V, Cristini V, Li KC, et al. Computational modeling of 3D tumor growth and angiogenesis for chemotherapy evaluation. PloS one. 2014;9(1):e83962.
10. Stéphanou A, Lesart A-C, Deverchère J, Juhem A, Popov A, Estève F. How tumour-induced vascular changes alter angiogenesis: Insights from a computational model. Journal of theoretical biology. 2017;419:211-26.
11. Vavourakis V, Wijeratne PA, Shipley R, Loizidou M, Stylianopoulos T, Hawkes DJ. A validated multiscale in-silico model for mechano-sensitive tumour angiogenesis and growth. PLoS computational biology. 2017;13(1):e1005259.
12. Shamsi M, Saghafian M, Dejam M, Sanati-Nezhad A. Mathematical modeling of the function of Warburg effect in tumor microenvironment. Scientific reports. 2018;8(1):1-13.
13. Nikmaneshi MR, Firoozabadi B, Mozafari A, Munn LL. A multi-scale model for determining the effects of pathophysiology and metabolic disorders on tumor growth. Scientific reports. 2020;10(1):1-20.
14. Baxter LT, Jain RK. Transport of fluid and macromolecules in tumors. I. Role of interstitial pressure and convection. Microvascular research. 1989;37(1):77-104.
15. Baxter LT, Jain RK. Transport of fluid and macromolecules in tumors. II. Role of heterogeneous perfusion and lymphatics. Microvascular research. 1990;40(2):246-63.
16. Baxter LT, Jain RK. Transport of fluid and macromolecules in tumors: III. Role of binding and metabolism. Microvascular research. 1991;41(1):5-23.
17. Baxter LT, Jain RK. Transport of fluid and macromolecules in tumors. IV. A microscopic model of the perivascular distribution. Microvascular research. 1991;41(2):252-72.
18. Jain RK. Vascular and interstitial barriers to delivery of therapeutic agents in tumors. Cancer and Metastasis Reviews. 1990;9(3):253-66.

19. Gevertz JL, Torquato S. Modeling the effects of vasculature evolution on early brain tumor growth. Journal of Theoretical Biology. 2006;243(4):517-31.
20. Baffert F, Thurston G, Rochon-Duck M, Le T, Brekken R, McDonald DM. Age-related changes in vascular endothelial growth factor dependency and angiopoietin-1-induced plasticity of adult blood vessels. Circulation research. 2004;94(7):984-92.
21. Carmeliet P. Angiogenesis in health and disease. Nature medicine. 2003;9(6):653.
22. Berk A, Zipursky S, Lodish H. Molecular Cell Biology 4th edition. National Center for Biotechnology InformationÕs Bookshelf; 2000.
23. Buchwald P. FEM-based oxygen consumption and cell viability models for avascular pancreatic islets. Theoretical Biology and Medical Modelling. 2009;6(1):5.
24. DeBerardinis RJ, Lum JJ, Hatzivassiliou G, Thompson CB. The biology of cancer: metabolic reprogramming fuels cell growth and proliferation. Cell metabolism. 2008;7(1):11-20.
25. Skog S, Tribukait B, Sundius G. Energy metabolism and ATP turnover time during the cell cycle of Ehrlich ascites tumour cells. Experimental cell research. 1982;141(1):23-9.
26. del Toro R, Prahst C, Mathivet T, Siegfried G, Kaminker JS, Larrivee B, et al. Identification and functional analysis of endothelial tip cell-enriched genes. Blood. 2010:blood-2010-02-270819.
27. Jakobsson L, Franco CA, Bentley K, Collins RT, Ponsioen B, Aspalter IM, et al. Endothelial cells dynamically compete for the tip cell position during angiogenic sprouting. Nature cell biology. 2010;12(10):943.
28. Kim M-C, Silberberg YR, Abeyaratne R, Kamm RD, Asada HH. Computational modeling of three-dimensional ECM-rigidity sensing to guide directed cell migration. Proceedings of the National Academy of Sciences. 2018;115(3):E390-E9.
29. Wong BW, Marsch E, Treps L, Baes M, Carmeliet P. Endothelial cell metabolism in health and disease: impact of hypoxia. The EMBO journal. 2017;36(15):2187-203.
30. Wood LB, Ge R, Kamm RD, Asada HH. Nascent vessel elongation rate is inversely related to diameter in in vitro angiogenesis. Integrative Biology. 2012;4(9):1081-9.
31. Eichmann A, Simons M. VEGF signaling inside vascular endothelial cells and beyond. Current opinion in cell biology. 2012;24(2):188-93.
32. Polacheck WJ, Charest JL, Kamm RD. Interstitial flow influences direction of tumor cell migration through competing mechanisms. Proceedings of the National Academy of Sciences. 2011;108(27):11115-20.
33. Haessler U, Teo JC, Foretay D, Renaud P, Swartz MA. Migration dynamics of breast cancer cells in a tunable 3D interstitial flow chamber. Integrative Biology. 2011;4(4):401-9.
34. Nikmaneshi MR, Firoozabadi B, Mozafari A. Chemo-Mechanistic multi-scale model of a three-dimensional tumor microenvironment to quantify chemotherapy response of cancer. Biotechnology and Bioengineering. 2021.
35. Cameron MA, Davis AL. A Mathematical Model of Angiogenesis in Glioblastoma Multiforme. 2009.
36. Nakatsu MN, Sainson RC, Pérez-del-Pulgar S, Aoto JN, Aitkenhead M, Taylor KL, et al. VEGF 121 and VEGF 165 regulate blood vessel diameter through vascular endothelial growth factor receptor 2 in an in vitro angiogenesis model. Laboratory investigation. 2003;83(12):1873-85.
37. Netti PA, Roberge S, Boucher Y, Baxter LT, Jain RK. Effect of transvascular fluid exchange on pressure–flow relationship in tumors: a proposed mechanism for tumor blood flow heterogeneity. Microvascular research. 1996;52(1):27-46.
38. Cai Y, Zhang J, Li Z. Multi-scale mathematical modelling of tumour growth and microenvironments in anti-angiogenic therapy. Biomedical engineering online. 2016;15(2):155.
39. Baish JW, Netti PA, Jain RK. Transmural coupling of fluid flow in microcirculatory network and interstitium in tumors. Microvascular research. 1997;53(2):128-41.

40. Welter M, Rieger H. Interstitial fluid flow and drug delivery in vascularized tumors: a computational model. PloS one. 2013;8(8):e70395.
41. Bates D, Hillman N, Pocock T, Neal C. Regulation of microvascular permeability by vascular endothelial growth factors. Journal of anatomy. 2002;200(5):523-34.
42. Cai Y, Wu J, Li Z, Long Q. Mathematical modelling of a brain tumour initiation and early development: a coupled model of glioblastoma growth, pre-existing vessel co-option, angiogenesis and blood perfusion. PloS one. 2016;11(3):e0150296.
43. DiResta GR, Nathan SS, Manoso MW, Casas-Ganem J, Wyatt C, Kubo T, et al. Cell proliferation of cultured human cancer cells are affected by the elevated tumor pressures that exist in vivo. Annals of biomedical engineering. 2005;33(9):1270-80.
44. Shirinifard A, Gens JS, Zaitlen BL, Popławski NJ, Swat M, Glazier JA. 3D multi-cell simulation of tumor growth and angiogenesis. PloS one. 2009;4(10):e7190.
45. Yapp DT, Wong MQ, Kyle AH, Valdez SM, Tso J, Yung A, et al. The differential effects of metronomic gemcitabine and antiangiogenic treatment in patient-derived xenografts of pancreatic cancer: treatment effects on metabolism, vascular function, cell proliferation, and tumor growth. Angiogenesis. 2016;19(2):229-44.
46. Turley RS, Fontanella AN, Padussis JC, Toshimitsu H, Tokuhisa Y, Cho EH, et al. Bevacizumab-induced alterations in vascular permeability and drug delivery: a novel approach to augment regional chemotherapy for in-transit melanoma. Clinical Cancer Research. 2012;18(12):3328-39.
47. Sengupta S, Eavarone D, Capila I, Zhao G, Watson N, Kiziltepe T, et al. Temporal targeting of tumour cells and neovasculature with a nanoscale delivery system. Nature. 2005;436(7050):568-72.
48. Zhou K, Zhang J-w, Wang Q-z, Liu W-y, Liu J-l, Yao L, et al. Apatinib, a selective VEGFR2 inhibitor, improves the delivery of chemotherapeutic agents to tumors by normalizing tumor vessels in LoVo colon cancer xenograft mice. Acta Pharmacologica Sinica. 2019;40(4):556-62.
49. Anderson AR. A hybrid mathematical model of solid tumour invasion: the importance of cell adhesion. Mathematical medicine and biology: a journal of the IMA. 2005;22(2):163-86.
50. Jain RK. Transport of molecules in the tumor interstitium: a review. Cancer research. 1987;47(12):3039-51.
51. Laursen T, Kirk J. Diffusion coefficients of carbon dioxide and glucose for a connective tissue membrane from individuals of various ages. Journal of gerontology. 1955;10(3):303-5.
52. Bao Z, Chen K, Krepel S, Tang P, Gong W, Zhang M, et al. High glucose promotes human glioblastoma cell growth by increasing the expression and function of chemoattractant and growth factor receptors. Translational oncology. 2019;12(9):1155-63.
53. Higgins C. Parameters that reflect the carbon dioxide content of blood. Parameters. 2008.
54. Anderson AR, Chaplain M. Continuous and discrete mathematical models of tumor-induced angiogenesis. Bulletin of mathematical biology. 1998;60(5):857-99.
55. Baldwin ME, Catimel B, Nice EC, Roufail S, Hall NE, Stenvers KL, et al. The specificity of receptor binding by vascular endothelial growth factor-d is different in mouse and man. Journal of Biological Chemistry. 2001;276(22):19166-71.
56. McIlhenny C, George W, Doughty J. A comparison of serum and plasma levels of vascular endothelial growth factor during the menstrual cycle in healthy female volunteers. British journal of cancer. 2002;86(11):1786-9.
57. Billy F, Ribba B, Saut O, Morre-Trouilhet H, Colin T, Bresch D, et al. A pharmacologically based multiscale mathematical model of angiogenesis and its use in investigating the efficacy of a new cancer treatment strategy. Journal of theoretical biology. 2009;260(4):545-62.
58. Davis S, Aldrich TH, Jones PF, Acheson A, Compton DL, Jain V, et al. Isolation of angiopoietin-1, a ligand for the TIE2 receptor, by secretion-trap expression cloning. Cell. 1996;87(7):1161-9.

59. Longstaff C. Plasminogen activation on the cell surface. Front Biosci. 2002;7:d244-d55.
60. Maisonpierre PC, Suri C, Jones PF, Bartunkova S, Wiegand SJ, Radziejewski C, et al. Angiopoietin-2, a natural antagonist for Tie2 that disrupts in vivo angiogenesis. Science. 1997;277(5322):55-60.
61. Plank M, Sleeman B, Jones P. A mathematical model of tumour angiogenesis, regulated by vascular endothelial growth factor and the angiopoietins. Journal of theoretical biology. 2004;229(4):435-54.
62. Engin H, Üstündağ Y, Tekin İÖ, Gökmen A. Plasma concentrations of Ang-1, Ang-2 and Tie-2 in gastric cancer. European cytokine network. 2012;23(1):21-4.
63. Cai Y, Xu S, Wu J, Long Q. Coupled modelling of tumour angiogenesis, tumour growth and blood perfusion. Journal of Theoretical Biology. 2011;279(1):90-101.
64. Masuhara K, Nakai T, Yamaguchi K, Yamasaki S, Sasaguri Y. Significant increases in serum and plasma concentrations of matrix metalloproteinases 3 and 9 in patients with rapidly destructive osteoarthritis of the hip. Arthritis & Rheumatism. 2002;46(10):2625-31.
65. Woodley-Cook J, Shin LY, Swystun L, Caruso S, Beaudin S, Liaw PC. Effects of the chemotherapeutic agent doxorubicin on the protein C anticoagulant pathway. Molecular cancer therapeutics. 2006;5(12):3303-11.
66. Pries A, Secomb T, Gaehtgens P. Structural adaptation and stability of microvascular networks: theory and simulations. American Journal of Physiology-Heart and Circulatory Physiology. 1998;275(2):H349-H60.
67. Stéphanou A, McDougall SR, Anderson AR, Chaplain MA. Mathematical modelling of the influence of blood rheological properties upon adaptative tumour-induced angiogenesis. Mathematical and Computer Modelling. 2006;44(1-2):96-123.
68. Stylianopoulos T, Jain RK. Combining two strategies to improve perfusion and drug delivery in solid tumors. Proceedings of the National Academy of Sciences. 2013;110(46):18632-7.
69. Pluen A, Boucher Y, Ramanujan S, McKee TD, Gohongi T, di Tomaso E, et al. Role of tumor–host interactions in interstitial diffusion of macromolecules: cranial vs. subcutaneous tumors. Proceedings of the National Academy of Sciences. 2001;98(8):4628-33.
70. Kitajima K, Fukuoka M, Kobayashi S, Kusunoki Y, Takada M, Negoro S, et al. Studies on the appropriate administration of cisplatin based on pharmacokinetics and toxicity. Gan to Kagaku Ryoho Cancer & Chemotherapy. 1987;14(8):2517-23.
71. Gaudreault J, Bruno R, Kabbinavar F, Sing A, Johnson D, Lu J. Clinical pharmacokinetics of bevacizumab following every 2-or every 3-week dosing. Journal of Clinical Oncology. 2004;22(14_suppl):3041-.
